# ﻿Ralph W. Holzenthal – a mentor and friend retires

**DOI:** 10.3897/zookeys.1111.83120

**Published:** 2022-07-11

**Authors:** Steffen U. Pauls, Robin Thomson, Ernesto Rázuri-Gonzales

**Affiliations:** 1 Senckenberg Research Institute and Natural History Museum Frankfurt, Senckenberganlage 25, 60325 Frankfurt, Germany Senckenberg Research Institute and Natural History Museum Frankfurt Frankfurt Germany; 2 Institute of Insect Biotechnology, Justus-Liebig-University Gießen, Heinrich-Buff-Ring 26, 35392 Gießen, Germany Justus-Liebig-University Gießen Gießen Germany; 3 University of Minnesota Insect Collection, Department of Entomology, University of Minnesota, St Paul, MN, USA University of Minnesota St. Paul United States of America

## ﻿A much too brief CV

Ralph Holzenthal began his studies in caddisfly diversity at the University of New Orleans, where he completed his Masters of Science degree in 1980 after exploring the caddisfly of southeastern Louisiana and southern Mississippi, which was in his own words, “as far as I could travel in a day with a non-existing budget”. Together with the research of Steve Harris and Paul Lago, Ralph’s MSc thesis significantly contributed to advancing our knowledge of caddisflies in the Southeastern United States (Harris et al. 1982; Holzenthal et al. 1982; Lago et al. 1982). After considering several topics and advisors, Ralph joined John Morse at Clemson University for his Ph.D. on Neotropical Leptoceridae. Specifically, he assessed and analyzed the diversity, evolution, and biogeography of Neotropical Leptoceridae and revised their systematics. At Clemson he also met Steve Hamilton. Ralph and ‘both Steves’ developed very productive professional relationships and personal friendships that last through today. Thus, Ralph became part of a line of Trichoptera workers that would significantly impact our knowledge of the Trichoptera fauna of North and South America. First and foremost, this line goes back to Herbert Ross, Fernand Schmid, Glenn Wiggins, Oliver Flint, and John Morse.

During his graduate studies, Ralph attended his first International Symposium of Trichoptera at Clemson in 1983 and presented and published the first paper of his Ph.D. work in the symposium’s proceedings: a description of the new genus *Achoropsyche* as the first contribution in the nine-part “Studies in Neotropical Leptoceridae” series (Holzenthal 1984). Perhaps more importantly he began many long-term relationships and friendships. He met Fernand Schmid of the Ministry of Agriculture Canada whose style and diligence in the preparation of Trichoptera he idolized and which Ralph has since passed on to generations of students. Through the Symposium he also intensified his interactions with Oliver Flint, an entomologist from the Smithsonian National Museum of Natural History, who became an important mentor for Ralph and whose collected material was the basis for a large part of Ralph’s dissertation.

He completed his Ph.D. in Entomology at Clemson University in 1985. Ralph’s Neotropical work then really took off when he received three National Science Foundation (NSF) grants to work on the caddisflies of Costa Rica. He began the first grant as a postdoctoral researcher at Clemson University in 1986 but was offered a faculty position as Faculty Director of the University of Minnesota Insect Collection (UMSP) at the University of Minnesota in spring of the same year. The grants and the work on Trichoptera of Costa Rica were instrumental to his tenure at UMSP. His six months sabbatical in 1997–1998 as visiting professor at the Universidade Federal do Paraná, Curitiba, Brazil was crucial to expanding his network in South America and broadening the taxonomic and regional scope of his work. The second influential grant to his career was a 2001 NSF “Partnerships for Enhancing Expertise in Taxonomy” (PEET) award. This grant, focused on formally training students in Trichoptera systematics and taxonomy, enabled Ralph to pursue one of his scientific career passions: training students from across the Americas in insect taxonomy, systematics, and biodiversity. He has since trained numerous younger colleagues through formal and informal avenues, thereby building a legacy of excellent Trichoptera taxonomists particularly known for their revisionary studies and excellent illustrations. Beyond teaching “standard” courses of an entomological curriculum, he also developed unique courses on scientific illustration of insects. These are sought after globally, and Ralph has been invited to give numerous workshops around the world to students and professionals. Ralph also excelled at communicating his knowledge with students. He was a cherished teacher and won multiple faculty teaching awards at the University of Minnesota. These included the FAME Award (Faculty Award for Mentorship in Entomology) presented through “Frenatae”, the University of Minnesota’s Entomology Graduate Student Organization in 2005 and 2010, which highlights how highly valued Ralph was as a teacher and mentor by his students. Many of his former mentees submitted articles to this volume, to show their gratitude and respect for Ralph’s life work. The topics he thus influenced range from faunal surveys and checklists (Cavalcante-Silva et al. 2022; Chuluunbat et al. 2022; Houghton 2022; Luna-Luna et al. 2022) and ecological studies (Houghton et al. 2022; Ríos-Touma et al. 2022) to descriptive taxonomy (Bueno-Soria et al. 2022; Cavalcante-Silva et al. 2022; Martins et al. 2022; Pereira et al. 2022; Ramírez-Carmona et al. 2022; Rázuri-Gonzales et al. 2022) and systematic revisions (Blahnik and Andersen 2022; Sganga et al. 2022; Thomson et al. 2022a, b).

**Table 1.** Students mentored by R.W. Holzenthal (in reverse chronological order).

M.Sc.	Ph.D.
Heather Cummins, 2014	Luis Ernesto Rázuri-Gonzales, 2020
Joel Gardner, 2013 (Co-Advisor with M. Spivak)	Lucas Marques de Carmargos, 2020
Anne M. Wasmund, 2006	Robin Thomson, 2014
Dianne M. Crane, 1994 (Co-Advisor with R.D. Moon)	Desiree R. Robertson, 2010
Margot P. Monson, 1994	Maria Lourdes Chamorro, 2009
Roger J. Blahnik, 1991	Henrique Paprocki, 2008
Roger M. Strand, 1991	Fernando Muñoz Quesada, 2003
	David Houghton, 2002
	Aysha Prather, 2002
	Roger J. Blahnik, 1996
	Atilano Contreras-Ramos, 1996
	Sonia M.N. Lazzari, 1990

**Figure 1.**
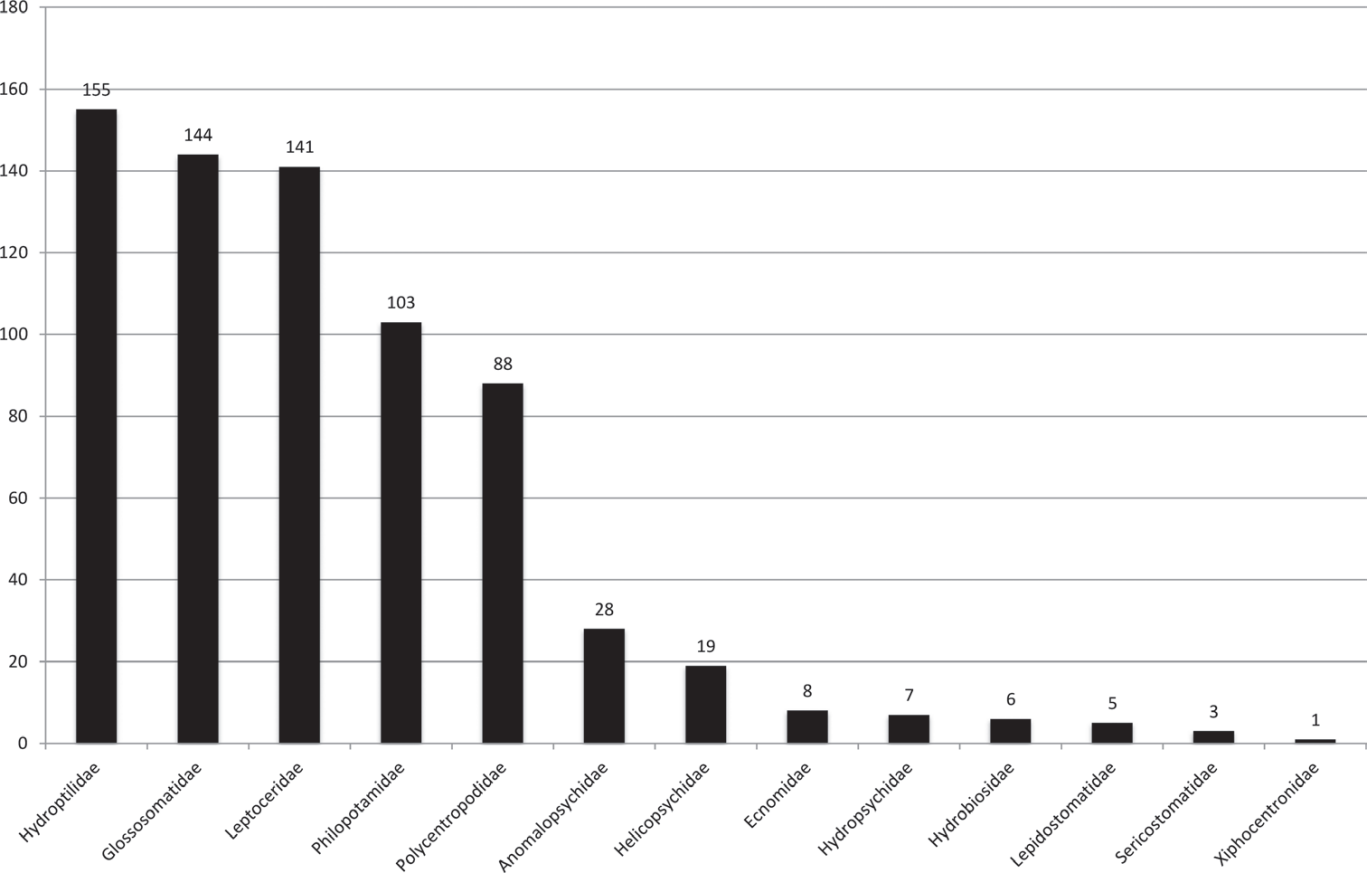
Number of new species described by R.W. Holzenthal by family (ordered by rank).

**Figure 2.**
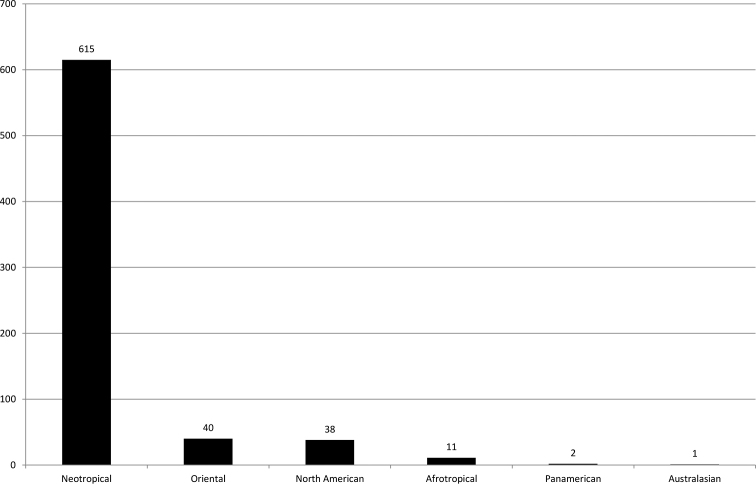
Number of species described by R.W. Holzenthal by biogeographical region (ordered by rank).

The impact of his scientific work on Neotropical and other Trichoptera is seen in the 708 species (Figs 1, 2) he has described in dozens of publications and compendia (e.g., Flint et al. 1999; Holzenthal and Calor 2017), the twelve new genera and subgenera described, the incredible collection he has built, and the many students he trained.

## ﻿Our interpretation of the scientific legacy of Ralph W. Holzenthal

Ralph’s impact goes beyond pure numbers; his taxonomic contributions are characterized by particularly comprehensive treatments of morphology and species descriptions, often representing extensive revisions of taxonomic groups (e.g., Holzenthal 1982; Holzenthal and Flint 1995) or regional faunas (e.g., Andersen and Holzenthal 2001, 2002) and authoritative catalogues (Flint et al. 1999; Holzenthal and Calor 2017). This resulted in a body of work of lasting value that is still relevant today, decades later, and is used not only as a basis for identifications but also for further systematic work.

In our opinion, and this was passed on to us by Ralph’s mentoring, excellent taxonomic publications not only clarify the morphological characters that are central to the identification of the taxa in question but also allow interpretation of homology of characters relevant for evolutionary analysis. Excellent and clear illustrations are central for this purpose. Ralph has developed his own style for traditionally and digitally inked line drawings and illustrations that succeed in being both unambiguous, thereby allowing identification, but also being sufficiently detailed to allow initial assessments of homology and convergence. He has introduced these methods to many subsequent generations through formal classes and informal courses and workshops.

Another cornerstone in Ralph’s career has been an openness to innovative methodologies. Ralph conducted phylogenetic analyses to clarify evolutionary questions early in his doctoral studies, initially following the principles established by Hennig (1969). Hennigian phylogenetics were a major innovation in taxonomy at the time of Ralph’s first studies, influencing his decision to work with John Morse for his Ph.D., as John Morse was already using these methods. Throughout his career Ralph applied many methodological innovations to his work. These included digital approaches to illustration (Holzenthal 2008), database systems for collections management, and computer-generated natural language descriptions and taxonomic keys (e.g., DELTA; Dallwitz 1974, 1980). He later adopted the use of molecular data in phylogenetic systematics and developed them further in cooperation with colleagues (Kjer et al. 2001), up to current applications of genomics in phylogenetics (Thomson et al. 2022b). As Faculty Director of the University of Minnesota Insect Collection (UMSP), he leaves behind one of the foremost Neotropical Trichoptera collections in the world, and a well-curated insect collection of more than 4 million specimens where species level identification lies at an astonishing ~ 70%. All Trichoptera specimens are databased and have machine-readable barcode labels (for a searchable database of these specimens, see https://scan-bugs.org/portal/). This approach to taxonomy and systematics, not only for its own sake but also as a service to other scientists, is what we believe sets Ralph’s contributions apart.

Ralph also has a strong awareness of obstacles to taxonomic publication and the central role of identification keys in organismal biology. This awareness led Ralph to join ZooKeys as a Trichoptera subject editor, where for five years his editorial leadership influenced many authors of excellent taxonomic papers. This is a further example of his service-oriented mindset.

But to what end did Ralph make these contributions? By making the world’s caddisfly fauna more available to systematists as well as evolutionary and conservation biologists, Ralph has helped advance the taxonomy of Trichoptera beyond the pioneering efforts of Dr. Oliver S. Flint to a new stage of knowledge for the Neotropics. In addition to descriptive and revisionary taxonomy, he has also advocated for the value of museums and collections in an ongoing and uphill endeavor. In the face of global climate change, this is an enterprise that now seems more important than ever because the secure refuges set aside to protect species no longer seem so permanent or secure. Natural habitats have been disappearing at alarming rates for the last few decades, which impacts our livelihoods and welfare. Biological inventories in turn raise awareness of the benefits of protecting these habitats and the biodiversity they hold. However, particularly in the tropics, these inventories usually focus on relatively well-known, easily identifiable, or charismatic groups such as birds, mammals, butterflies, and ants, while many other groups are scarcely known. By subsequently establishing protected areas known to be diverse, conservationists and biodiversity researchers aspire that these less well-known groups can also be protected and eventually described. At the rate natural habitats are being destroyed, however, it is unlikely the focus on few protected areas will suffice to preserve all the hitherto unknown diversity.

In a recent paper, Ralph and colleagues recorded 310 caddisfly species from Ecuador and estimated that only 54% of the Trichoptera fauna from this country is known to science (Ríos-Touma et al. 2017). Moreover, several Neotropical genera found in Ecuador are highly endemic at the species level (e.g., *Amphoropsyche*, *Atanatolica*, and *Contulma*), with new genera and species routinely discovered and described in these areas (Holzenthal et al. 2017, 2018). The same scenario applies in the forests of other tropical countries, and estimating how many additional species may have initially been present before these forests were deforested is impossible to accomplish.

Beyond diversity predictions and reserving natural habitats in the hope of protecting diversity, a fundamental concern for the loss of species should begin with a sense of urgency to know what might be lost. Because species are not really fathomable until they are more than theoretical numbers. How can one truly mourn the loss of species that were not even known to exist? How will one know that they were truly lost if there are no records that they existed in the first place? How do we still have such a poor knowledge of our planet’s biodiversity after more than 250 years naming species? If the diversity of species on the planet is as great as scientists have estimated, why does the description of biodiversity of the planet receive such a low priority?

Ralph understands that providing empirical evidence and a comprehensible characterization of biological diversity is of utmost importance to supporting conservation efforts. After a stellar career in taxonomy, with the description of hitherto 708 species previously unknown to science (see Figs 1, 2), Ralph can join the ranks of the many thousands of taxonomists who have contributed to the description of the Earth’s biological diversity. Many of his descriptions were conducted with students he trained, thereby ensuring a lasting legacy of his work and his ideas on high-quality approaches to taxonomy that will transcend through generations.

Over the years, Ralph’s thinking and his approach to taxonomy have inspired many others, especially young scientists and students, as is evident in the articles in this issue. Perhaps more importantly, all his students maintain a love and appreciation of both taxonomy and the diversity of aquatic insects. Ralph’s knowledge and enthusiasm for these topics has impressed his students to such a degree that they all maintained subject and mentor close to their hearts.

## ﻿List of publications

**1980**

Poirrier MA, Holzenthal RW (1980) Records of spongilla-flies [Neuroptera: Sisyridae] from Mississippi. Journal of the Mississippi Academy of Science 25: 1–2.

**1982**

Harris SC, Lago PK, Holzenthal RW (1982) An annotated checklist of the caddisflies (Trichoptera) of Mississippi and Southeastern Louisiana. Part II: Rhyacophiloidea. Proceedings of the Entomological Society of Washington 84: 509–512.

Holzenthal RW (1982) The caddisfly genus *Setodes* in North America (Trichoptera: Leptoceridae). Journal of the Kansas Entomological Society 55: 253–271.

Holzenthal RW, Harris SC, Lago PK (1982) An annotated checklist of the caddisflies (Trichoptera) of Mississippi and southeastern Louisiana. Part III: Limnephiloidea and conclusions. Proceedings of the Entomological Society of Washington 84: 513–520.

Lago PK, Holzenthal RW, Harris SC (1982) An annotated checklist of the caddisflies (Trichoptera) of Mississippi and Southeastern Louisiana. Part I: Introduction and Hydropsychoidea. Proceedings of the Entomological Society of Washington 84: 495–508.

**1983**

Holzenthal RW, Kelley RW (1983) New micro-caddisflies from the southeastern United States (Trichoptera: Hydroptilidae). Florida Entomologist 66: 464–472.

**1984**

Hamilton SW, Holzenthal RW (1984) The caddisfly genus *Helicopsyche* in America, north of Mexico (Trichoptera: Helicopsychidae) (abstract). In: Morse JC (Ed.) Proceedings of the 4^th^ International Symposium on Trichoptera. Dr. W. Junk, The Hague, 167 pp.

Holzenthal RW (1984) Studies in Neotropical Leptoceridae (Trichoptera) I: *Achoropsyche*, a new genus. In: Morse JC (Ed.) Proceedings of the 4^th^ International Symposium on Trichoptera. Dr. W. Junk, The Hague, 181–184.

Morse JC, Holzenthal RW (1984) Trichoptera genera. In: Merritt RW, Cummins KW (Eds) An Introduction to the Aquatic Insects of North America, 2^nd^ Edn. Kendall-Hunt, Dubuque, 312–347.

**1985**

Bart Jr JL, Holzenthal RW (1985) Feeding ecology of *Necturusbeyeri* in Louisiana. Journal of Herpetology 19: 402–410.

Holzenthal RW (1985) Studies in Neotropical Leptoceridae (Trichoptera) II: *Amphoropsyche*, a new genus and species of Leptocerinae from northern South America. International Journal of Entomology 27: 255–269.

Holzenthal RW (1985) Studies in Neotropical Leptoceridae (Trichoptera): their diversity, evolution, and biogeography, with revisions of selected genera. Doctoral, Clemson University, South Carolina.

Holzenthal RW, Harris SC (1985) The female of *Setodesguttatus* with distribution notes (Trichoptera: Leptoceridae). Journal of the Kansas Entomological Society 58: 166–167.

**1986**

Bueno-Soria J, Holzenthal RW (1986) Estudios de insectos acuáticos V: descripción de tres nuevas especies de trichópteros de México: (Trichoptera: Philopotamidae). Anales del Instituto de Biología, Universidad Nacional Autónoma de México, Serie Zoología 57: 137–144.

Hamilton SW, Holzenthal RW (1986) Two new species of caddisflies from Georgia (Trichoptera: Polycentropodidae, Hydroptilidae). Proceedings of the Entomological Society of Washington 88: 163–166.

Holzenthal RW (1986) The Neotropical species of *Notalina*, a southern group of long-horned caddisflies (Trichoptera: Leptoceridae). Systematic Entomology 11: 61–73.

Holzenthal RW (1986) Studies in Neotropical Leptoceridae (Trichoptera), IV: a revision of *Brachysetodes* Schmid. Transactions of the American Entomological Society 111: 407–440.

Holzenthal RW (1986) Studies in Neotropical Leptoceridae (Trichoptera), a new species of *Amphoropsyche*, with a redescription of the immature stages of *A.insularis* (Flint). Annals of the Entomological Society of America 79: 251–255.

Holzenthal RW (1986) Studies in Neotropical Leptoceridae (Trichoptera), VI: immature stages of *Hudsonemaflaminii* (Navas) and the evolution and historical biogeography of Hudsonemini (Triplectininae). Proceedings of the Entomological Society of Washington 88: 268–279.

**1987**

Morse JC, Holzenthal RW (1987) Higher classification of Triplectidinae (Trichoptera: Leptoceridae). In: Bournaud M, Tachet H (Eds) Proceedings of the 5^th^ International Symposium on Trichoptera. Dr. W. Junk, Dordrecht, The Netherlands, 139–144.

**1988**

Holzenthal RW (1988) Systematics of Neotropical *Triplectides* (Trichoptera: Leptoceridae). Annals of the Entomological Society of America 81: 187–208.

Holzenthal RW (1988) Studies in Neotropical Leptoceridae (Trichoptera), VIII: the genera *Atanatolica* Mosely and *Grumichella* Müller (Triplectidinae: Grumichellini). Transactions of the American Entomological Society 114: 71–128.

Holzenthal RW, Hamilton SW (1988) New species and records of Costa Rican *Polycentropus* (Trichoptera: Polycentropodidae). Journal of the New York Entomological Society 96: 332–344.

Monson MP, Holzenthal RW, Ahlstrand GG (1988) The larvae and pupa of *Cochiliopsychevazquezae* (Trichoptera: Helicopsychidae). Journal of the North American Benthological Society 7: 152–159.

Holzenthal RW (1988) Catálogo sistemático de los Trichopteros de Costa Rica (Insecta: Trichoptera). Brenesia 29: 51–82.

**1989**

Holzenthal RW (1989) Studies in Neotropical Leptoceridae (Trichoptera), IX: a new genus and species from southeastern Brazil. Aquatic Insects 11: 29–32.

Harris SC, Holzenthal RW (1990) Hydroptilidae (Trichoptera) from Costa Rica: the genus *Mayatrichia* Mosely. Journal of the New York Entomological Society 98: 453–460.

**1991**

Holzenthal RW (1991) A preliminary assessment of the fauna of the Reserva Forestal de San Ramón, Costa Rica. In: Ortiz RV (Ed.) Memoria de investigación Reserva Forestal de San Ramón. Universidad de Costa Rica, San Ramón, 79–81.

Holzenthal RW, Harris SC (1991) The larva of *Byrsopteryxmirifica* Flint, with an assessment of the phylogenetic placement of the genus within the Leuchotrichiini (Trichoptera: Hydroptilidae). In: Tomaszewski C (Ed.) Proceedings of the 6^th^ International Symposium on Trichoptera. Adam Mickiewicz University Press, Poznan, Poland, 403–407.

**1992**

Blahnik RJ, Holzenthal RW (1992) Revision of the Neotropical genus *Chimarrhodella* Lestage (Trichoptera: Philopotamidae). Systematic Entomology 17: 109–132.

Blahnik RJ, Holzenthal RW (1992) New species of ChimarrasubgenusChimarra Stephens from Costa Rica (Trichoptera: Philopotamidae). Proceedings of the Entomological Society of Washington 94: 409–438.

Holzenthal RW, Harris SC (1992) Hydroptilidae (Trichoptera) of Costa Rica: the genus *Oxyethira* Eaton. Journal of the New York Entomological Society 100: 155–177.

Holzenthal RW, Strand RM (1992) New species of *Lepidostoma* from Mexico and Central America (Trichoptera: Lepidostomatidae). Proceedings of the Entomological Society of Washington 94: 490–499.

**1993**

Harris SC, Holzenthal RW (1993) Phylogeny of the species groups of *Alisotrichia*, *sensu lato*, with the description of a new species from Costa Rica (Trichoptera: Hydroptilidae). In: Otto C (Ed.) Proceedings of the 7^th^ International Symposium on Trichoptera. Backhuys Publishers, Leiden, The Netherlands, 155–160.

Monson MP, Holzenthal RW (1993) A new species and new records of *Oxyethira* (Trichoptera: Hydroptilidae) from Minnesota. Journal of the North American Benthological Society 12: 438–443.

Muñoz-Quesada F, Holzenthal RW (1993) New species and records of Costa Rican *Austrotinodes* (Trichoptera: Ecnomidae). Proceedings of the Entomological Society of Washington 95: 564–573.

**1994**

Harris SC, Holzenthal RW (1994) Hydroptilidae (Trichoptera) of Costa Rica and the Neotropics: Systematics of the genus *Byrsopteryx* Flint (Stactobiini). Journal of the New York Entomological Society 102: 154–192.

**1995**

Holzenthal RW (1995) The caddisfly genus *Nectopsyche*: new *gemma* group species from Costa Rica and the Neotropics (Trichoptera: Leptoceridae). Journal of the North American Benthological Society 14: 61–83.

Holzenthal RW, Blahnik RJ (1995) New species of Smicridea (Rhyacophylax) (Trichoptera: Hydropsychidae) from Costa Rica. Entomological News 106: 213–223.

Holzenthal RW, Flint Jr OS (1995) Studies of Neotropical caddisflies, LI: systematics of the Neotropical caddisfly genus *Contulma* (Trichoptera: Anomalopsychidae). Smithsonian Contributions to Zoology 575: 1–59.

**1996**

Morse JC, Holzenthal RW (1996) Trichoptera genera. In: Merritt RW, Cummins KW (Eds) An Introduction to the Aquatic Insects of North America, 3^rd^ Edn. Kendall/Hunt Publishing Company, Dubuque, USA, 350–386.

**1997**

Harris SC, Holzenthal RW (1997) *Mejicanotrichia*, a new genus of microcaddisflies from Mexico and Guatemala (Trichoptera: Hydroptilidae). In: Holzenthal RW, Flint Jr OS (Eds) Proceedings of the 8^th^ International Symposium on Trichoptera. Ohio Biological Survey, Columbus, 123–128.

Holzenthal RW (1997) The caddisfly (Trichoptera) family Atriplectididae in the Neotropics. In: Holzenthal RW, Flint Jr OS (Eds) Proceedings of the 8^th^ International Symposium on Trichoptera. Ohio Biological Survey, Columbus, 157–165.

Holzenthal RW, Flint J, O.S. (Eds) (1997) Proceedings of the 8^th^ International Symposium on Trichoptera. Ohio Biological Survey, Columbus, 496 pp.

Muñoz-Quesada F, Holzenthal RW (1997) A new species of Xiphocentron (Antillotrichia) from Costa Rica with semiterrestrial immature stages (Trichoptera: Xiphocentronidae). In: Holzenthal RW, Flint Jr OS (Eds) Proceedings of the 8^th^ International Symposium on Trichoptera. Ohio Biological Survey, Columbus, 355–363.

**1998**

Bueno-Soria J, Holzenthal RW (1998) Studies in aquatic insects XIV: descriptions of eight new species of *Ochrotrichia* Mosely (Trichoptera: Hydroptilidae) from Costa Rica. Proceedings of the Biological Society of Washington 111: 604–612.

**1999**

Andersen T, Holzenthal RW (1999) The genus *Allosetodes* Banks, 1931, a junior synonym of *Triaenodes* MacLachlan, 1865 (Trichoptera: Leptoceridae). In: Malicky H, Chantaramongkol P (Eds) Proceedings of the 9^th^ International Symposium on Trichoptera. Faculty of Science, Chiang Mai University, Chiang Mai, Thailand, 7–16.

Flint Jr OS, Holzenthal RW, Harris SC (1999) Nomenclatural and systematic changes in the Neotropical caddisflies. Insecta Mundi 13: 73–84.

Flint Jr OS, Holzenthal RW, Harris SC (1999) Catalog of the Neotropical Caddisflies (Trichoptera). Special Publication, Ohio Biological Survey, Columbus, Ohio, 239 pp.

Harris SC, Holzenthal RW (1999) Hydroptilidae (Trichoptera) of Costa Rica: the genus *Hydroptila* Dalman. Studies on Neotropical Fauna and Environment 34: 16–51.

Holzenthal RW, Harris SC (1999) The genus *Costatrichia* Mosely in Costa Rica, with a review of the Neotropical species (Trichoptera: Hydroptilidae). Proceedings of the Entomological Society of Washington 101: 540–568.

Luhman JC, Holzenthal RW, Kjaerandsen JK (1999) New host record of a ceraphronid (Hymenoptera) in Trichoptera pupae. Journal of Hymenoptera Research 8: 126.

**2001**

Andersen T, Holzenthal RW (2001) West African *Triaenodes* McLachlan (Trichoptera: Leptoceridae). 1. Introduction and subgenus Triaenodella Mosely. Tijdschrift voor Entomologie 144: 225–246.

Houghton DC, Holzenthal RW, Monson MP, MacLean DB (2001) Updated checklist of the Minnesota caddisflies (Tricoptera [Trichoptera]) with geographic affinities. Transactions of the American Entomological Society 127: 495–512

Kjer KM, Blahnik RJ, Holzenthal RW (2001) Phylogeny of Trichoptera (Caddisflies): Characterization of signal and noise within multiple datasets. Systematic Biology 50: 781–816. https://doi.org/10.1080/106351501753462812

**2002**

Andersen T, Holzenthal RW (2002) West African *Adicella* McLachlan, 1877 (Trichoptera: Leptoceridae). Proceedings of the 10^th^ International Symposium on Trichoptera, Postdam (Germany), July – August 2000. Deutsches Entomologisches Institut, Goecke & Evers, Keltern, 88–95.

Andersen T, Holzenthal RW (2002) *Adicellasyriaca* Ulmer, 1907 not occurring in the Afrotropical region (Trichoptera: Leptoceridae). Aquatic Insects 24: 161–164. https://doi.org/10.1163/22119434-900000100

Andersen T, Holzenthal RW (2002) West African *Triaenodes* McLachlan (Trichoptera: Leptoceridae). 2. Subgenus Triaenodes sensu stricto. Tijdschrift voor Entomologie 145: 61–88. https://doi.org/10.1163/22119434-900000100

Harris SC, Flint Jr OS, Holzenthal RW (2002) Two new genera of Hydroptilidae from the neotropics (Trichoptera: Hydroptilidae: Stactobiini). Journal of the New York Entomological Society 110: 49–64.

Harris SC, Flint Jr OS, Holzenthal RW (2002) Review of the Neotropical genus *Flintiella* (Trichoptera: Hydroptilidae: Stactobiini). Journal of the New York Entomological Society 110: 65–90. https://doi.org/10.1664/0028-7199(2002)110[0065:ROTNGF]2.0.CO;2

Harris SC, Holzenthal RW, Flint Jr OS (2002) Review of the Neotropical genus *Bredinia* (Trichoptera: Hydroptilidae: Stactobiini). Annals of Carnegie Museum 71: 13–45.

Holzenthal RW, Cressa C (2002) The Trichoptera, caddisflies, of Venezuela: Three new species and records of *Atopsyche* Banks (Hydrobiosidae). Studies on Neotropical Fauna and Environment 37: 133–143. https://doi.org/10.1076/snfe.37.2.133.8578

Holzenthal RW, Harris SC (2002) New species of *Nothotrichia* Flint (Trichoptera: Hydroptilidae) from Brazil and Costa Rica. Proceedings of the Entomological Society of Washington 104: 106–110.

Kjer KM, Blahnik RJ, Holzenthal RW (2002) Phylogeny of caddisflies (Insecta, Trichoptera). Zoologica Scripta 31: 83–91. https://doi.org/10.1046/j.0300-3256.2001.00079.x

Paprocki H, Holzenthal RW (2002) A review of the Brazilian genus *Barypenthus* Burmeister (Trichoptera: Odontoceridae). Proceedings of the 10^th^ International Symposium on Trichoptera), Postdam (Germany), July – August 2000. Deutsches Entomologisches Institut, Goecke & Evers, Keltern, 223–230.

Prather AL, Holzenthal RW (2002) The identity of *Silvataresexcelsus* Navás, 1931. In: Mey W (Ed.) Proceedings of the 10^th^ International Symposium on Trichoptera, Postdam (Germany), July – August 2000. Deutsches Entomologisches Institut, Goecke & Evers, Keltern, 231–234.

**2003**

Bueno-Soria J, Holzenthal RW (2003) New species and records of the microcaddisfly genus *Metrichia* Ross from Costa Rica (Trichoptera: Hydroptilidae). Studies on Neotropical Fauna and Environment 38: 173–197. https://doi.org/10.1076/snfe.38.3.173.28164

Holzenthal RW, de Almeida GL (2003) New species of Polycentropodidae (Trichoptera) from southeastern and southern Brazil. Proceedings of the Entomological Society of Washington 105: 22–29.

Houghton DC, Holzenthal RW (2003) Updated conservation status of protected Minnesota caddisflies. Great Lakes Entomologist 36: 35–40.

Kjer KM, Holzenthal RW, Blahnik RJ (2003) Phylogeny of Trichoptera. Entomologische Abhandlungen 61: 166.

Paprocki H, Holzenthal RW, Cressa C (2003) A new species of *Smicridea* McLachlan (Trichoptera: Hydropsychidae) from Venezuela and its role in travertine biogenesis. Journal of the North American Benthological Society 22: 401–409.

**2004**

Blahnik RJ, Holzenthal RW (2004) Collection and curation of Trichoptera, with an emphasis on pinned material. Nectopsyche, Neotropical Trichoptera Newsletter 1: 8–20 http://hdl.handle.net/11299/190744

Blahnik RJ, Paprocki H, Holzenthal RW (2004) New distribution and species records of Trichoptera from southern and southeastern Brazil. Biota Neotropica 4: 1–6. https://doi.org/10.1590/S1676-06032004000100009

Bueno-Soria J, Holzenthal RW (2004) New species of the genus *Ochrotrichia* Mosely (Trichoptera: Hydroptilidae) from Mexico and Panama. Transactions of the American Entomological Society 130: 245–269.

Chamorro-Lacayo ML, Holzenthal RW (2004) Seven new species of *Polyplectropus* Ulmer (Trichoptera: Polycentropodidae) from Costa Rica. Proceedings of the Entomological Society of Washington 106: 202–216.

Holzenthal RW (2004) Three new species of Chilean caddisflies (Insecta: Trichoptera). Proceedings of the Entomological Society of Washington 106: 110–117.

Holzenthal RW (2004) Essential resources for research on the Neotropical Trichoptera fauna. Nectopsyche, Neotropical Trichotpera Newsletter 2: 7–9.

Holzenthal RW, Andersen T (2004) The caddisfly genus *Triaenodes* in the Neotropics (Trichoptera: Leptoceridae). Zootaxa 511: 1–80. https://doi.org/10.11646/zootaxa.511.1.1

Holzenthal RW, Pes AMO (2004) A new genus of long-horned caddisfly from the Amazon basin (Trichoptera: Leptoceridae: Grumichellini). Zootaxa 621: 1–16. https://doi.org/10.11646/zootaxa.621.1.1

Johanson KA, Holzenthal RW (2004) Thirteen new species and new distribution records of Helicopsyche (Feropsyche) Johanson from Venezuela (Trichoptera: Helicopsychidae). Zootaxa 711: 1–40. https://doi.org/10.11646/zootaxa.711.1.1

Paprocki H, Holzenthal RW, Blahnik RJ (2004) Checklist of the Trichoptera (Insecta) of Brazil I. Biota Neotropica 4: 1–22. https://doi.org/10.1590/S1676-06032004000100008

**2005**

Hamilton SW, Holzenthal RW (2005) Five new species of Polycentropodidae (Trichoptera) from Ecuador and Venezuela. Zootaxa 810: 1–14. https://doi.org/10.11646/zootaxa.810.1.1

Robertson DR, Holzenthal RW (2005) The Neotropical caddisfly genus *Tolhuaca* (Trichoptera: Glossosomatidae). Zootaxa 1063: 53–68. https://doi.org/10.11646/zootaxa.1063.1.3

**2006**

Blahnik RJ, Holzenthal RW (2006) Revision of the genus *Culoptila* (Trichoptera: Glossosomatidae). Zootaxa 1233: 1–52. https://doi.org/10.11646/zootaxa.1233.1.1

Calor AR, Holzenthal RW, Amorim DS (2006) Phylogenetic analysis of Notalina (Neonotalina) Holzenthal (Trichoptera: Leptoceridae), with the description of two new species from southeastern Brazil. Zootaxa 1131: 33–48. https://doi.org/10.11646/zootaxa.1131.1.2

Holzenthal RW (2006) Book review: Caddisflies the underwater architects. Journal of the North American Benthological Society 25: 263–265. https://doi.org/10.1899/0887-3593(2006)25[263:br]2.0.co;2

Holzenthal RW (2006) Caddisflies, the underwater architects [book review]. American Entomologist 52: 199–200. https://doi.org/10.1093/ae/52.3.199

Holzenthal RW, Blahnik RJ (2006) The caddisfly genus *Protoptila* in Costa Rica (Trichoptera: Glossosomatidae). Zootaxa 1197: 1–37. https://doi.org/10.11646/zootaxa.1197.1.1

Holzenthal RW, Robertson DR (2006) Four new species of *Contulma* from South America (Trichoptera: Anomalopsychidae). Zootaxa 1355: 49–59. https://doi.org/10.11747/zootaxa.1355.1.3

Robertson DR, Holzenthal RW (2006) The Neotropical caddisfly genus *Canoptila* (Trichoptera: Glossosomatidae). Zootaxa 1272: 45–59. https://doi.org/10.11646/zootaxa.1272.1.2

**2007**

Blahnik RJ, Holzenthal RW, Prather AL (2007) The lactic acid method for clearing Trichoptera genitalia. In: Bueno-Soria J, Barba-Álvarez R, Armitage BJ (Eds) Proceedings of the 12^th^ International Symposium on Trichoptera, Mexico City (Mexico), June 2006. The Caddis Press, Columbus, 9–14.

Chamorro-Lacayo ML, Maes J-M, Holzenthal RW, Blahnik RJ (2007) Updated checklist of the Trichoptera of Nicaragua. In: Bueno-Soria J, Barba-Álvarez R, Armitage BJ (Eds) Proceedings of the 12^th^ International Symposium on Trichoptera, Mexico City (Mexico), June 2006. The Caddis Press, Columbus, 37–50.

Holzenthal RW, Andersen T (2007) Review of the caddisfly genus *Tagalopsyche* with the description of new species and a related new genus (Trichoptera: Leptoceridae: Mystacidini). Zootaxa 1483: 1–32. https://doi.org/10.11646/zootaxa.1483.1.1

Holzenthal RW, Blahnik RJ, Kjer KM, Prather AP (2007) An update on the phylogeny of caddisflies (Trichoptera). In: Bueno-Soria J, Barba-Alvarez R, Armitage B (Eds) Proceedings of the 12^th^ International Symposium on Trichoptera, Mexico City (Mexico), June 2006. The Caddis Press, Columbus, 143–153.

Holzenthal RW, Blahnik RJ, Prather AL, Kjer KM (2007) Order Trichoptera Kirby, 1813 (Insecta), caddisflies. Zootaxa 1668: 639–698. https://doi.org/10.11646/zootaxa.1668.1.29

Wasmund AM, Holzenthal RW (2007) A revision of the Neotropical caddisfly genus *Rhyacopsyche*, with the description of 13 new species (Trichoptera: Hydroptilidae). Zootaxa 1634: 1–59. https://doi.org/10.11646/zootaxa.1634.1.1

**2008**

Blahnik RJ, Holzenthal RW (2008) Revision of the Mexican and Central American species of *Mortoniella* (Trichoptera: Glossosomatidae: Protoptilinae). Zootaxa 1711: 1–72. https://doi.org/10.11646/zootaxa.1711.1.1

Bueno-Soria J, Holzenthal RW (2008) The genus *Ochrotrichia* Mosely (Trichoptera: Hydroptilidae) in Costa Rica, with the description of four new species. Zootaxa 1763: 41–54. https://doi.org/10.11646/zootaxa.1763.1.3

Calor AR, Holzenthal RW (2008) Phylogeny of Grumichellini Morse, 1981 (Trichoptera: Leptoceridae) with the description of a new genus from southeastern Peru. Aquatic Insects 30: 245–259. https://doi.org/10.1080/01650420802334087

Holzenthal RW (2008) Digital illustration of insects. American Entomologist 54: 218–221. https://doi.org/10.1093/ae/54.4.218

Holzenthal RW, Prather AL, Marshall SA (2008) Interactive key to the aquatic insect orders of North America, CD-ROM. In: Merritt RW, Cummins KW, Berg MA (Eds) An Introduction to the Aquatic Insects of North America, 4^th^ Edn. Kendall/Hunt, Dubuque, USA.

Morse JC, Holzenthal RW (2008) Chapter 18, Caddisfly genera. In: Merritt RW, Cummins KW, Berg MA (Eds) An Introduction to the Aquatic Insects of North America, 4^th^ Edn. Kendall/Hunt, Dubuque, USA, 481–552.

Muñoz-Quesada FJ, Holzenthal RW (2008) Revision of the Nearctic species of the caddisfly genus *Wormaldia* McLachlan (Trichoptera: Philopotamidae). Zootaxa 1838: 1–75. https://doi.org/10.11646/zootaxa.3998.1.1

Robertson DR, Holzenthal RW (2008) Two new species and a new record of *Protoptila* from Bolivia (Trichoptera: Glossosomatidae: Protoptilinae). Annals of the Entomological Society of America 101: 465–473. https://doi.org/10.1603/0013-8746(2008)101[465:TNSAAN]2.0.CO;2

**2009**

Blahnik RJ, Holzenthal RW, Huisman J (2009) *Chimarra* of Sabah and Sarawak, northern Borneo (Trichoptera: Philopotamidae). Tijdschrift voor Entomologie 152: 109–166. https://doi.org/10.1163/22119434-900000272.

Holzenthal RW (2009) Trichoptera (Caddisflies). In: Likens GE (Ed.) Encyclopedia of Inland Waters Volume 2. Elsevier, Oxford, UK, 456–467.

**2010**

Chamorro ML, Holzenthal RW (2010) Taxonomy and phylogeny of New World *Polyplectropus* Ulmer, 1905 (Trichoptera: Psychomyioidea: Polycentropodidae) with the description of 39 new species. Zootaxa 2582: 1–252. https://doi.org/10.11646/zootaxa.2582.1.1

Holzenthal RW, Blahnik RJ (2010) Systematics of the Neotropical caddisfly genus *Notidobiella* Schmid (Trichoptera, Sericostomatidae), with the description of 3 new species. ZooKeys 71: 23–47. https://doi.org/10.3897/zookeys.71.791

Holzenthal RW, Robertson DR, Pauls SU, Mendez PK (2010) Taxonomy and systematics: contributions to benthology and J-NABS. Journal of the North American Benthological Society 29: 147–169. https://doi.org/10.1899/08-065.1

Houghton DC, Holzenthal RW (2010) Historical and contemporary biological diversity of Minnesota caddisflies: a case study of landscape-level species loss and trophic composition shift. Journal of the North American Benthological Society 29: 480–495. https://doi.org/10.1899/09-029.1

Johanson KA, Holzenthal RW (2010) The snail-case caddisfly subgenus Helicopsyche (Feropsyche) in Costa Rica, with the description of 3 new species (Trichoptera: Helicopsychidae). Zootaxa 2689: 37–47. https://doi.org/10.11646/zootaxa.2689.1.4

Pauls SU, Blahnik RJ, Zhou X, Wardwell CT, Holzenthal RW (2010) DNA barcode data confirm new species and reveal cryptic diversity in Chilean Smicridea (Smicridea) (Trichoptera: Hydropsychidae). Journal of the North American Benthological Society 29: 1058–1074. https://doi.org/10.1899/09-108.1

Pauls SU, Holzenthal RW, Ngera MF (2010) Two new species of *Triaenodes* McLachlan 1865 from streams in the Lake Kivu basin, South Kivu, Democratic Republic of the Congo (Trichoptera, Leptoceridae). Denisia 29: 277–285.

Thomson RE, Holzenthal RW (2010) New Neotropical species of the genus *Austrotinodes* Schmid (Trichoptera: Ecnomidae). Zootaxa 2347: 38–50. https://doi.org/10.11646/zootaxa.2437.1.2

**2011**

Blahnik RJ, Holzenthal RW (2011) Revision of the austral South American species of *Mortoniella* (Trichoptera: Glossosomatidae: Protoptilinae). Zootaxa 2851: 1–75. https://doi.org/10.11636/zootaxa.2851.1.1

Chamorro ML, Holzenthal RW (2011) Phylogeny of Polycentropodidae Ulmer, 1903 (Trichoptera: Annulipalpia: Psychomyioidea) inferred from larval, pupal and adult characters. Invertebrate Systematics 25: 219–253. https://doi.org/10.1071/IS10024

Hamilton SW, Holzenthal RW (2011) Twenty-four new species of *Polycentropus* (Trichoptera, Polycentropodidae) from Brazil. ZooKeys 76: 1–53. https://doi.org/10.3897/zookeys.76.790

Holzenthal RW, Morse JC, Kjer KM (2011) Order Trichoptera Kirby, 1813. In: Zhang Z-Q (Ed.) Animal biodiversity: An outline of higher-level classification and survey of taxonomic richness. Zootaxa 3148: 209–211. https://doi.org/10.11646/zootaxa.3148.1.40

Holzenthal RW, Rázuri-Gonzales LE (2011) A new species of *Amphoropsyche* (Trichoptera, Leptoceridae) from Ecuador, with a key to the species in the genus. ZooKeys 211: 59–65. https://doi.org/10.3897/zookeys.111.813

Mendez PK, Holzenthal RW, Steiner JWH (2011) The Trichoptera Literature Database: a collaborative bibliographic resource for world caddisfly research. In: Majecka K, Majecki J, Morse J (Eds) Proceedings of the 13^th^ International Symposium on Trichoptera, Białowieża (Poland), June 2009. Magnolia Press, Auckland, NZ, 331–337. https://doi.org/10.11646/zoosymposia.5.1.25

Robertson DR, Holzenthal RW (2011) Revision of the Neotropical caddisfly genus *Itauara* Müller, 1888 (Trichoptera, Glossosomatidae). ZooKeys 114: 41–100. https://doi.org/10.3897/zookeys.114.1405

**2012**

Blahnik RJ, Holzenthal RW (2012) New Neotropical species of *Chimarra* (Trichoptera, Philopotamidae). ZooKeys 184: 1–33. https://doi.org/10.3897/zookeys.184.2911

Holzenthal RW, Ríos-Touma B (2012) *Contulmapaluguillensis* (Trichoptera: Anomalopsychidae), a new caddisfly from the high Andes of Ecuador, and its natural history. Freshwater Science 31: 442–450. https://doi.org/10.1899/11-067.1

Santos APM, Holzenthal RW (2012) Three new species of *Atopsyche* Banks (Trichoptera, Hydrobiosidae) from Brazil. ZooKeys 207: 65–78.

Thomson RE, Holzenthal RW (2012) New species and records of Hydroptilidae (Trichoptera) from Venezuela. ZooKeys 185: 19–39. https://doi.org/10.3897/zookeys.185.2909

**2013**

Robertson DR, Holzenthal RW (2013) Revision and phylogeny of the caddisfly subfamily Protoptilinae (Trichoptera: Glossosomatidae) inferred from adult morphology and mitochondrial DNA. Zootaxa 3723: 1–99. https://doi.org/10.11646/zootaxa.3723.1.1

Vshivkova TS, Flint OS, Holzenthal RW, Kjer KM, Frandsen PB, Thomson RE, Egorov AB (2013) First data on Trichoptera fauna (Insecta) of streams and ponds of Vostok Bay Basin (Peter the Great Bay, Primorye Territory). In: Makarchenko EA (Ed.) Freshwater Life. Dalnauka, Vladivostok, Russia, 123–143.

**2014**

Blahnik RJ, Holzenthal RW (2014) Review and redescription of species in the *Oecetisavara* group, with the description of 15 new species (Trichoptera, Leptoceridae). ZooKeys: 1–83. https://doi.org/10.3897/zookeys.376.6047

**2015**

Armitage BJ, Harris SC, Holzenthal RW (2015) The Trichoptera of Panama I. New records for caddisflies (Insecta: Trichoptera) from the Republic of Panama. Insecta Mundi 0435: 1–10.

Holzenthal RW, Thomson RE, Ríos-Touma B (2015) Order Trichoptera. In: Thorp JH, Rogers DC (Eds) Ecology and General Biology, Vol I: Thorp and Covich’s Freshwater Invertebrates, 4^th^ Edn. Academic Press, XXXX, 965–1002. https://doi.org/10.1016/b978-0-12-385026-3.00038-3

Muñoz-Quesada FJ, Holzenthal RW (2015) Revision of the Neotropical species of the caddisfly genus *Wormaldia* McLachlan (Trichoptera: Philopotamidae). Zootaxa 3998: 1–138. https://doi.org/10.11646/zootaxa.3998.1.1

Thomson RE, Holzenthal RW (2015) A revision of the Neotropical caddisfly genus *Leucotrichia* Mosely, 1934 (Hydroptilidae, Leucotrichiinae). ZooKeys 499: 1–100. https://doi.org/10.3897/zookeys.499.8360

**2016**

Calor AR, Holzenthal RW, Froehlich CG (2016) Phylogeny and revision of the Neotropical genus *Grumichella* Müller (Trichoptera: Leptoceridae), including nine new species and a key. Zoological Journal of the Linnean Society 176: 137–169. https://doi.org/10.1111/zoj.12310

Holzenthal R, Ríos-Touma B (2016) A new Ecuadorian species of the rare Neotropical caddisfly genus *Amphoropsyche* Holzenthal (Trichoptera, Leptoceridae). ZooKeys 640: 59–67. https://doi.org/10.3897/zookeys.640.10344

Holzenthal RW, Blahnik RJ, Calor AR (2016) Three new species of *Helicopsyche* von Siebold (Trichoptera: Helicopsychidae) from Brazil. Zootaxa 4078: 344–353. https://doi.org/10.11646/zootaxa.4078.1.29

Kjer KM, Thomas JA, Zhou X, Frandsen PB, Scott E, Holzenthal RW (2016) Progress on the phylogeny of caddisflies (Trichoptera). In: Vshivkova T, Morse JC (Eds) Proceedings of the 14^th^ International Symposium on Trichoptera, Vladivostok (Russia), July 2012. Magnolia Press, Auckland, NZ, 248–256. https://doi.org/10.11646/zoosymposia.10.1.23

Rázuri-Gonzales E, Holzenthal RW (2016) New synonyms in the highly diverse caddisfly genus *Smicridea* (Trichoptera, Hydropsychidae). ZooKeys 637: 21–31. https://doi.org/10.3897/zookeys.637.10148

Vshivkova T, Flint O, Ito T, Ivanov V, Holzenthal R, Melnitsky S, Mey W, Nozaki T, Oh MW, Drozdov K, Tojo K, Saito R, Tori T (2016) The List of Caddisflies (Insecta, Trichoptera) collected in South Primorye during the symposium and post-symposium excursions of the XIV International Symposium on Trichoptera (5 and 8–13 July 2012). In: Vshivkova TS, Morse JC (Eds) Proceedings of the 14^th^ International Symposium on Trichoptera, Vladivostok (Russia), July 2012. Magnolia Press, Auckland, NZ, 64–84. https://doi.org/10.11646/zoosymposia.10.1.7

Zhou X, Frandsen PB, Holzenthal RW, Beet CR, Bennett KR, Blahnik RJ, Bonada N, Cartwright D, Chuluunbat S, Cocks GV, Collins GE, deWaard J, Dean J, Flint OS, Hausmann A, Hendrich L, Hess M, Hogg ID, Kondratieff BC, Malicky H, Milton MA, Morinière J, Morse JC, Mwangi FN, Pauls SU, Gonzalez MR, Rinne A, Robinson JL, Salokannel J, Shackleton M, Smith B, Stamatakis A, StClair R, Thomas JA, Zamora-Muñoz C, Ziesmann T, Kjer KM (2016) The Trichoptera barcode initiative: a strategy for generating a species-level Tree of Life. Philosophical Transactions of the Royal Society of London B: Biological Sciences 371: 1–11. https://doi.org/10.1098/rstb.2016.0025

**2017**

Blahnik RJ, Holzenthal RW (2017) Revision of the northern South American species of *Mortoniella* Ulmer,1906 (Trichoptera: Glossosomatidae: Protoptilinae). Insecta Mundi 0602: 1–250.

Holzenthal RW, Calor AR (2017) Catalog of the Neotropical Trichoptera (Caddisflies). ZooKeys 654: 1–566. https://doi.org/10.3897/zookeys.654.9516

Holzenthal RW, Ríos-Touma B, Rázuri-Gonzales E (2017) New species of the endemic Neotropical caddisfly genus *Contulma* from the Andes of Ecuador (Trichoptera: Anomalopsychidae). PeerJ 5: e3967. https://doi.org/10.7717/peerj.3967

Quinteiro FB, Holzenthal RW (2017) Fourteen new species of *Oecetis* McLachlan, 1877 (Trichoptera: Leptoceridae) from the Neotropical region. PeerJ 5: e3753. https://doi.org/10.7717/peerj.3753

Rázuri-Gonzales E, Holzenthal RW, Ríos-Touma B (2017) Two new species of the rare Neotropical caddisfly genus *Amphoropsyche* Holzenthal (Trichoptera, Leptoceridae). ZooKeys 707: 63–72. https://doi.org/10.3897/zookeys.707.20759

Ríos-Touma B, Holzenthal RW, Huisman J, Thomson R, Rázuri-Gonzales E (2017) Diversity and distribution of the Caddisflies (Insecta: Trichoptera) of Ecuador. PeerJ 5: e2851. https://doi.org/10.7717/peerj.2851

**2018**

Barcelos-Silva P, Pes AM, Andrade-Souza V, Holzenthal RW (2018) Associating larvae and adults of the Neotropical caddisfly genus *Synoestropsis* Ulmer (Trichoptera: Hydropsychidae) using morphology and DNA mitochondrial sequences. Zoologischer Anzeiger 277: 169–189. https://doi.org/10.1016/j.jcz.2018.08.002

Holzenthal R, Blahnik RJ, Ríos-Touma B (2018) New species and a new genus of Philopotamidae from the Andes of Bolivia and Ecuador (Insecta, Trichoptera). ZooKeys 780: 89–108. https://doi.org/10.3897/zookeys.780.26977

Holzenthal RW, Ríos-Touma B (2018) Nectopsyche of Ecuador: a new species from the high Andean páramo and redescription of Nectopsyche spiloma (Ross) (Trichoptera: Leptoceridae). PeerJ 6: e4981. https://doi.org/10.7717/peerj.4981

Pes AM, Holzenthal RW, Sganga JV, Santos APM, Barcelos-Silva P, Camargos LM (2018) Order Trichoptera. In: Hamada N, Thorp JH, Rogers DC (Eds) Keys to Neotropical Hexapoda, Thorp and Covich’s Freshwater Invertebrates - Volume III, 4^th^ Ed. Academic Press, London, 237–324. https://doi.org/10.1016/B978-0-12-804223-6.00010-X

Rázuri-Gonzales E, Holzenthal RW, Ríos-Touma B (2018) New *Atanatolica* species from Ecuador (Trichoptera, Leptoceridae). ZooKeys 793: 97–114. https://doi.org/10.3897/zookeys.793.26712

**2019**

Ngera MF, Pauls SU, Holzenthal RW, Bagalwa M, Bisimwa MA, Mushayuma EM, and Cammaerts DR. 2019. Contribution to the knowledge of the macroinvertebrate fauna of the streams of Kahuzi-Biega National Park, Democratic Republic of Congo. African Journal of Aquatic Science 44: 127–142. https://doi.org/10.2989/16085914.2019.1598840

**2020**

Thomas JA, Frandsen PB, Prendini E, Zhou X, Holzenthal RW (2020) A multigene phylogeny and timeline for Trichoptera (Insecta). Systematic Entomology 45: 670–686. https://doi.org/10.1111/syen.12422

Vilarino A, Holzenthal RW (2020) Systematic revision of the caddisfly genus *Machairocentron* Schmid (Trichoptera: Psychomyioidea: Xiphocentronidae). Insect Systematics & Evolution 52: 375–411. https://doi.org/10.1163/1876312X-bja10013

**2021**

Olsen LK, Heckenhauer J, Sproul JS, Dikow RB, Gonzalez VL, Kweskin MP, Taylor AM, Wilson SB, Stewart RJ, Zhou X, Holzenthal R, Pauls SU, Frandsen PB (2021) Draft Genome Assemblies and Annotations of *Agrypniavestita* Walker, and *Hesperophylaxmagnus* Banks Reveal Substantial Repetitive Element Expansion in Tube Case-Making Caddisflies (Insecta: Trichoptera). Genome Biology and Evolution 13(3): evab013. https://doi.org/10.1093/gbe/evab013

Ríos-Touma B, Holzenthal RW, Rázuri-Gonzales E, Heckenhauer J, Pauls SU, Storer CG, Frandsen PB (2021) De Novo Genome Assembly and Annotation of an Andean Caddisfly, *Atopsychedavidsoni* Sykora, 1991, a Model for Genome Research of High-Elevation Adaptations. Genome Biology and Evolution 14(1): evab286. https://doi.org/10.1093/gbe/evab286

## ﻿New genera and subgenera circumscribed by R.W. Holzenthal

*Achoropsyche* Holzenthal, 1984

*Amazonatolica* Holzenthal & Pes, 2004

*Amphoropsyche* Holzenthal, 1985

*Aymaradella* Holzenthal, Blahnik & Ríos-Touma, 2018

*Fernandoschmidia* Holzenthal & Andersen, 2007

*Mejicanotrichia* Harris & Holzenthal, 1997

Mortoniella (Nanotrichia) Blahnik & Holzenthal, 2017

*Neoathripsodes* Holzenthal, 1989

Notalina (Neonotalina) Holzenthal, 1986

*Orinochotrichia* Harris, Flint & Holzenthal, 2002

*Osflintia* Calor & Holzenthal, 2008

*Tizatetrichia* Harris, Flint & Holzenthal, 2002

## ﻿New species described by R.W. Holzenthal (arranged alphabetically by family and species)

### Anomalopsychidae

*Contulmaadamsae* Holzenthal & Flint, 1995

*Contulmabacula* Holzenthal & Flint, 1995

*Contulmaboliviensis* Holzenthal & Robertson, 2006

*Contulmacaldensis* Holzenthal & Flint, 1995

*Contulmacataracta* Holzenthal & Flint, 1995

*Contulmacolombiensis* Holzenthal & Flint, 1991

*Contulmacostaricensis* Holzenthal & Flint, 1995

*Contulmaechinata* Holzenthal & Flint, 1995

*Contulmaecuadorensis* Holzenthal & Flint, 1995

*Contulmafluminensis* Holzenthal & Robertson, 2006

*Contulmainornata* Holzenthal & Flint, 1995

*Contulmalanceolata* Holzenthal & Flint, 1995

*Contulmalina* Holzenthal, Ríos-Touma & Rázuri-Gonzales, 2017

*Contulmameloi* Holzenthal & Robertson, 2006

*Contulmanevada* Holzenthal & Flint, 1995

*Contulmapaluguillensis* Holzenthal & Ríos-Touma, 2012

*Contulmapapallacta* Holzenthal & Flint, 1995

*Contulmapenai* Holzenthal & Flint, 1995

*Contulmaquito* Holzenthal, Ríos-Touma & Rázuri-Gonzales, 2017

*Contulmasancta* Holzenthal & Flint, 1995

*Contulmasangay* Holzenthal, Ríos-Touma & Rázuri-Gonzales, 2017

*Contulmaspinosa* Holzenthal & Flint, 1991

*Contulmatalamanca* Holzenthal & Flint, 1995

*Contulmatapanti* Holzenthal & Flint, 1995

*Contulmatica* Holzenthal & Flint, 1995

*Contulmatijuca* Holzenthal & Flint, 1995

*Contulmatripui* Holzenthal & Robertson, 2006

*Contulmavalverdei* Holzenthal & Flint, 1995

### Ecnomidae

*Austrotinodesabrachium* Thomson & Holzenthal, 2010

*Austrotinodesbelchioris* Thomson & Holzenthal, 2010

*Austrotinodesboliviensis* Thomson & Holzenthal, 2010

*Austrotinodescressae* Thomson & Holzenthal, 2010

*Austrotinodesdoublesi* Munioz-Quesada & Holzenthal, 1993

*Austrotinodesinbio* Munioz-Quesada & Holzenthal, 1993

*Austrotinodeslongispinum* Thomson & Holzenthal, 2010

*Austrotinodestaquaralis* Thomson & Holzenthal, 2010

### Glossosomatidae

*Canoptilawilliami* Robertson & Holzenthal, 2006

*Culoptilabidentata* Blahnik & Holzenthal, 2006

*Culoptilabuenoi* Blahnik & Holzenthal, 2006

*Culoptilacascada* Blahnik & Holzenthal, 2006

*Culoptilahamata* Blahnik & Holzenthal, 2006

*Culoptilapararusia* Blahnik & Holzenthal, 2006

*Culoptilaplummerensis* Blahnik & Holzenthal, 2006

*Culoptilatapanti* Blahnik & Holzenthal, 2006

*Culoptilaunispina* Blahnik & Holzenthal, 2006

*Culoptilavexillifera* Blahnik & Holzenthal, 2006

*Itauaraalexanderi* Robertson & Holzenthal, 2011

*Itauarabidentata* Robertson & Holzenthal, 2011

*Itauarablahniki* Robertson & Holzenthal, 2011

*Itauaracharlotta* Robertson & Holzenthal, 2011

*Itauaraemilia* Robertson & Holzenthal, 2011

*Itauaraflinti* Robertson & Holzenthal, 2011

*Itauaraguyanensis* Robertson & Holzenthal, 2011

*Itauarajamesii* Robertson & Holzenthal, 2011

*Itauarajulia* Robertson & Holzenthal, 2011

*Itauaralucinda* Robertson & Holzenthal, 2011

*Itauaraovis* Robertson & Holzenthal, 2011

*Itauaraperuensis* Robertson & Holzenthal, 2011

*Itauararodmani* Robertson & Holzenthal, 2011

*Itauarasimplex* Robertson & Holzenthal, 2011

*Itauaraspiralis* Robertson & Holzenthal, 2011

*Itauarastella* Robertson & Holzenthal, 2011

*Itauaratusci* Robertson & Holzenthal, 2011

*Itauaraunidentata* Robertson & Holzenthal, 2011

*Mastigoptilacomplicornuta* Holzenthal, 2004

*Mastigoptilaelae* Holzenthal, 2004

*Mortoniellaacauda* Blahnik & Holzenthal, 2011

*Mortoniellaacutiterga* Blahnik & Holzenthal, 2017

*Mortoniellaadamsae* Blahnik & Holzenthal, 2017

*Mortoniellaagosta* Blahnik & Holzenthal, 2011

*Mortoniellaakantha* Blahnik & Holzenthal, 2008

*Mortoniellaakrogeneios* Blahnik & Holzenthal, 2017

*Mortoniellaanakantha* Blahnik & Holzenthal, 2008

*Mortoniellaapplanata* Blahnik & Holzenthal, 2017

*Mortoniellaasymmetris* Blahnik & Holzenthal, 2011

*Mortoniellaauricularis* Blahnik & Holzenthal, 2017

*Mortoniellaaviceps* Blahnik & Holzenthal, 2008

*Mortoniellabarinasi* Blahnik & Holzenthal, 2017

*Mortoniellabiramosa* Blahnik & Holzenthal, 2017

*Mortoniellabocaina* Blahnik & Holzenthal, 2011

*Mortoniellabothrops* Blahnik & Holzenthal, 2017

*Mortoniellabrachyrhachos* Blahnik & Holzenthal, 2008

*Mortoniellabrevis* Blahnik & Holzenthal, 2017

*Mortoniellabuenoi* Blahnik & Holzenthal, 2008

*Mortoniellabulbosa* Blahnik & Holzenthal, 2017

*Mortoniellacarinula* Blahnik & Holzenthal, 2008

*Mortoniellacatherinae* Blahnik & Holzenthal, 2017

*Mortoniellacaudicula* Blahnik & Holzenthal, 2008

*Mortoniellachalalan* Blahnik & Holzenthal, 2017

*Mortoniellacognata* Blahnik & Holzenthal, 2017

*Mortoniellacoheni* Blahnik & Holzenthal, 2017

*Mortoniellacornuta* Blahnik & Holzenthal, 2017

*Mortoniellacrescentis* Blahnik & Holzenthal, 2011

*Mortoniellacressae* Blahnik & Holzenthal, 2017

*Mortoniellacroca* Blahnik & Holzenthal, 2017

*Mortoniellacurtispina* Blahnik & Holzenthal, 2017

*Mortoniellacurvistylus* Blahnik & Holzenthal, 2017

*Mortonielladentiterga* Blahnik & Holzenthal, 2017

*Mortonielladinotes* Blahnik & Holzenthal, 2017

*Mortonielladolonis* Blahnik & Holzenthal, 2011

*Mortonielladraconis* Blahnik & Holzenthal, 2017

*Mortoniellaemarginata* Blahnik & Holzenthal, 2017

*Mortoniellaesrossi* Blahnik & Holzenthal, 2017

*Mortoniellafalcicula* Blahnik & Holzenthal, 2008

*Mortoniellaflexuosa* Blahnik & Holzenthal, 2017

*Mortoniellafroehlichi* Blahnik & Holzenthal, 2011

*Mortoniellafurcula* Blahnik & Holzenthal, 2017

*Mortoniellagilli* Blahnik & Holzenthal, 2017

*Mortoniellagracilis* Blahnik & Holzenthal, 2017

*Mortoniellagrandiloba* Blahnik & Holzenthal, 2017

*Mortoniellaguahybae* Blahnik & Holzenthal, 2011

*Mortoniellaguyanensis* Blahnik & Holzenthal, 2017

*Mortoniellahamata* Blahnik & Holzenthal, 2017

*Mortoniellahystricosa* Blahnik & Holzenthal, 2011

*Mortoniellaintervales* Blahnik & Holzenthal, 2011

*Mortoniellalangleyae* Blahnik & Holzenthal, 2017

*Mortoniellalatispina* Blahnik & Holzenthal, 2011

*Mortoniellalicina* Blahnik & Holzenthal, 2017

*Mortoniellalongispina* Blahnik & Holzenthal, 2011

*Mortoniellalongiterga* Blahnik & Holzenthal, 2017

*Mortoniellameloi* Blahnik & Holzenthal, 2011

*Mortoniellamembranacea* Blahnik & Holzenthal, 2017

*Mortoniellamexicana* Blahnik & Holzenthal, 2008

*Mortoniellamonopodis* Blahnik & Holzenthal, 2017

*Mortoniellamunozi* Blahnik & Holzenthal, 2008

*Mortoniellaopinionis* Blahnik & Holzenthal, 2008

*Mortoniellapanamensis* Blahnik & Holzenthal, 2008

*Mortoniellapapillata* Blahnik & Holzenthal, 2008

*Mortoniellaparaguaiensis* Blahnik & Holzenthal, 2011

*Mortoniellaparameralda* Blahnik & Holzenthal, 2017

*Mortoniellaparauna* Blahnik & Holzenthal, 2011

*Mortoniellaparaunota* Blahnik & Holzenthal, 2011

*Mortoniellapaucispina* Blahnik & Holzenthal, 2017

*Mortoniellapectinella* Blahnik & Holzenthal, 2008

*Mortoniellapica* Blahnik & Holzenthal, 2017

*Mortoniellaproakantha* Blahnik & Holzenthal, 2017

*Mortoniellaprolata* Blahnik & Holzenthal, 2017

*Mortoniellapropinqua* Blahnik & Holzenthal, 2008

*Mortoniellapumila* Blahnik & Holzenthal, 2011

*Mortoniellapusilla* Blahnik & Holzenthal, 2011

*Mortoniellaquadridactyla* Blahnik & Holzenthal, 2017

*Mortoniellaquadrispina* Blahnik & Holzenthal, 2017

*Mortoniellarectiflexa* Blahnik & Holzenthal, 2017

*Mortoniellaredunca* Blahnik & Holzenthal, 2008

*Mortoniellarodmani* Blahnik & Holzenthal, 2008

*Mortoniellaruedae* Blahnik & Holzenthal, 2017

*Mortoniellaschlingeri* Blahnik & Holzenthal, 2017

*Mortoniellasicula* Blahnik & Holzenthal, 2008

*Mortoniellasilacea* Blahnik & Holzenthal, 2017

*Mortoniellasimplicis* Blahnik & Holzenthal, 2017

*Mortoniellasinuosa* Blahnik & Holzenthal, 2017

*Mortoniellaspangleri* Blahnik & Holzenthal, 2017

*Mortoniellaspatulata* Blahnik & Holzenthal, 2017

*Mortoniellastilula* Blahnik & Holzenthal, 2008

*Mortoniellatanyrhabdos* Blahnik & Holzenthal, 2017

*Mortoniellatapanti* Blahnik & Holzenthal, 2008

*Mortoniellataurina* Blahnik & Holzenthal, 2008

*Mortoniellatriangularis* Blahnik & Holzenthal, 2017

*Mortoniellatridens* Blahnik & Holzenthal, 2017

*Mortoniellatripuiensis* Blahnik & Holzenthal, 2011

*Mortoniellatriramosa* Blahnik & Holzenthal, 2017

*Mortoniellatruncata* Blahnik & Holzenthal, 2011

*Mortoniellatusci* Blahnik & Holzenthal, 2017

*Mortoniellaumbonata* Blahnik & Holzenthal, 2008

*Mortoniellauruguaiensis* Blahnik & Holzenthal, 2011

*Mortoniellavariabilis* Blahnik & Holzenthal, 2017

*Mortoniellavenezuelensis* Blahnik & Holzenthal, 2017

*Mortoniellazamora* Blahnik & Holzenthal, 2017

*Protoptilaaltura* Holzenthal & Blahnik, 2006

*Protoptilabribri* Holzenthal & Blahnik, 2006

*Protoptilachitaria* Holzenthal & Blahnik, 2006

*Protoptilacristula* Holzenthal & Blahnik, 2006

*Protoptiladiablita* Robertson & Holzenthal, 2008

*Protoptilajolandae* Holzenthal & Blahnik, 2006

*Protoptilajulieta* Robertson & Holzenthal, 2008

*Protoptilakjeri* Holzenthal & Blahnik, 2006

*Protoptilastrepsicera* Holzenthal & Blahnik, 2006

*Protoptilatrichoglossa* Holzenthal & Blahnik, 2006

*Tolhuacabrasiliensis* Robertson & Holzenthal, 2005

### Helicopsychidae

*Helicopsychealajuela* Johanson & Holzenthal, 2010

*Helicopsycheangeloi* Holzenthal, Blahnik & Calor, 2016

*Helicopsycheauroa* Johanson & Holzenthal, 2004

*Helicopsychecamuriensis* Johanson & Holzenthal, 2004

*Helicopsychecirculata* Johanson & Holzenthal, 2004

*Helicopsychedisjuncta* Johanson & Holzenthal, 2004

*Helicopsychedorsocurvata* Johanson & Holzenthal, 2010

*Helicopsychegolfitoensis* Johanson & Holzenthal, 2010

*Helicopsycheguara* Holzenthal, Blahnik & Calor, 2016

*Helicopsychelaneblina* Johanson & Holzenthal, 2004

*Helicopsychelara* Johanson & Holzenthal, 2004

*Helicopsychelazzariae* Holzenthal, Blahnik & Calor, 2016

*Helicopsychelinabena* Johanson & Holzenthal, 2004

*Helicopsycheneblinensis* Johanson & Holzenthal, 2004

*Helicopsycheperija* Johanson & Holzenthal, 2004

*Helicopsychesuccincta* Johanson & Holzenthal, 2004

*Helicopsychesucrensis* Johanson & Holzenthal, 2004

*Helicopsychetachira* Johanson & Holzenthal, 2004

*Helicopsychevenezuelensis* Johanson & Holzenthal, 2004

### Hydrobiosidae

*Atopsycheallani* Holzenthal & Cressa, 2002

*Atopsycheblahniki* Santos & Holzenthal, 2012

*Atopsychegalharada* Santos & Holzenthal, 2012

*Atopsycheparauna* Santos & Holzenthal, 2012

*Atopsycherinconi* Holzenthal & Cressa, 2002

*Atopsychesegninii* Holzenthal & Cressa, 2002

### Hydropsychidae

*Smicrideafigueroai* Holzenthal, 2004

*Smicridealourditae* Pauls, Blahnik, Zhou, Wardwell & Holzenthal, 2010

*Smicrideanemorosa* Holzenthal & Blahnik, 1995

*Smicrideapatinae* Pauls, Blahnik & Holzenthal, 2010

*Smicrideasingri* Holzenthal & Blahnik, 1995

*Smicrideatapanti* Holzenthal & Blahnik, 1995

*Smicrideatravertinera* Paprocki, Holzenthal & Cressa, 2003

### Hydroptilidae

*Alisotrichiatiza* Harris & Holzenthal, 1993

*Angrisanoiaotarosa* Wasmund & Holzenthal, 2007

*Angrisanoiashorti* Thomson & Holzenthal, 2012

*Brediniaalza* Harris, Holzenthal & Flint, 2002

*Brediniadavenporti* Harris, Holzenthal & Flint, 2002

*Brediniaemarginata* Harris, Holzenthal & Flint, 2002

*Brediniaespinosa* Harris, Holzenthal & Flint, 2002

*Brediniaguanacasteca* Harris, Holzenthal & Flint, 2002

*Brediniamanabiensis* Harris, Holzenthal & Flint, 2002

*Brediniamexicana* Harris, Holzenthal & Flint, 2002

*Brediniapilcopata* Harris, Holzenthal & Flint, 2002

*Brediniaselva* Harris, Holzenthal & Flint, 2002

*Brediniaspangleri* Harris, Holzenthal & Flint, 2002

*Brediniasucrensis* Harris, Holzenthal & Flint, 2002

*Brediniavenezuelensis* Harris, Holzenthal & Flint, 2002

*Brediniazulia* Harris, Holzenthal & Flint, 2002

*Byrsopteryxabrelata* Harris & Holzenthal, 1994

*Byrsopteryxchaconi* Harris & Holzenthal, 1994

*Byrsopteryxcuchilla* Harris & Holzenthal, 1994

*Byrsopteryxesparta* Harris & Holzenthal, 1994

*Byrsopteryxespinhosa* Harris & Holzenthal, 1994

*Byrsopteryxgomezi* Harris & Holzenthal, 1994

*Byrsopteryxloja* Harris & Holzenthal, 1994

*Byrsopteryxrayada* Harris & Holzenthal, 1994

*Byrsopteryxsolisi* Harris & Holzenthal, 1994

*Byrsopteryxtapanti* Harris & Holzenthal, 1994

*Byrsopteryxtica* Harris & Holzenthal, 1994

*Costatrichiacarara* Holzenthal & Harris, 1999

*Costatrichiacressae* Holzenthal & Harris, 1999

*Costatrichiaflinti* Holzenthal & Harris, 1999

*Flintiellaalajuela* Harris, Flint & Holzenthal, 2002

*Flintiellaastilla* Harris, Flint & Holzenthal, 2002

*Flintiellaboraceia* Harris, Flint & Holzenthal, 2002

*Flintiellaheredia* Harris, Flint & Holzenthal, 2002

*Flintiellapanamensis* Harris, Flint & Holzenthal, 2002

*Flintiellapizotensis* Harris, Flint & Holzenthal, 2002

*Flintiellatamaulipasa* Harris, Flint & Holzenthal, 2002

*Flintiellayanamona* Harris, Flint & Holzenthal, 2002

*Hydroptilacarara* Harris & Holzenthal, 1999

*Hydroptilacarolae* Holzenthal & Kelley, 1983

*Hydroptilacressae* Thomson & Holzenthal, 2012

*Hydroptiladisgalera* Holzenthal & Kelley, 1983

*Hydroptilamaritza* Harris & Holzenthal, 1999

*Hydroptilamaza* Harris & Holzenthal, 1999

*Hydroptilanusagandia* Harris & Holzenthal, 1999

*Hydroptilaosa* Harris & Holzenthal, 1999

*Hydroptilaouachita* Holzenthal & Kelley, 1983

*Hydroptilaparadenza* Harris & Holzenthal, 1999

*Hydroptilapoirrieri* Holzenthal & Kelley, 1983

*Hydroptilarastrilla* Harris & Holzenthal, 1999

*Hydroptilaroberta* Hamilton & Holzenthal, 1984

*Hydroptilasingri* Harris & Holzenthal, 1999

*Hydroptilatridentata* Holzenthal & Kelley, 1983

*Leucotrichiaangelinae* Thomson & Holzenthal, 2015

*Leucotrichiadenticulata* Thomson & Holzenthal, 2015

*Leucotrichiadianeae* Thomson & Holzenthal, 2015

*Leucotrichiafulminea* Thomson & Holzenthal, 2015

*Leucotrichiahispida* Thomson & Holzenthal, 2015

*Leucotrichiakateae* Thomson & Holzenthal, 2015

*Leucotrichiapectinata* Thomson & Holzenthal, 2015

*Leucotrichiarepanda* Thomson & Holzenthal, 2015

*Leucotrichiarhomba* Thomson & Holzenthal, 2015

*Leucotrichiariostoumae* Thomson & Holzenthal, 2015

*Leucotrichiasidneyi* Thomson & Holzenthal, 2015

*Leucotrichiatapantia* Thomson & Holzenthal, 2015

*Leucotrichiazopilote* Holzenthal & Harris, 1999

*Mayatrichiaillobia* Harris & Holzenthal, 1990

*Mejicanotrichiaestaquillosa* Harris & Holzenthal, 1997

*Metrichiaacicula* Bueno-Soria & Holzenthal, 2003

*Metrichiaalajuela* Bueno-Soria & Holzenthal, 2003

*Metrichiaamplitudinis* Bueno-Soria & Holzenthal, 2003

*Metrichiaancora* Bueno-Soria & Holzenthal, 2003

*Metrichiaangulosa* Bueno-Soria & Holzenthal, 2003

*Metrichiabostrychion* Thomson & Holzenthal, 2012

*Metrichiadecora* Bueno-Soria & Holzenthal, 2003

*Metrichiagordita* Bueno-Soria & Holzenthal, 2003

*Metrichialuna* Bueno-Soria & Holzenthal, 2003

*Metrichiamagna* Bueno-Soria & Holzenthal, 2003

*Metrichiamechuda* Bueno-Soria & Holzenthal, 2003

*Metrichiameta* Bueno-Soria & Holzenthal, 2003

*Metrichiapicuda* Bueno-Soria & Holzenthal, 2003

*Metrichiaprolata* Bueno-Soria & Holzenthal, 2003

*Metrichiapseudopatagonica* Bueno-Soria & Holzenthal, 2003

*Metrichiasavegra* Bueno-Soria & Holzenthal, 2003

*Metrichiaseparata* Bueno-Soria & Holzenthal, 2003

*Metrichiasesquipedalis* Bueno-Soria & Holzenthal, 2003

*Metrichiaspica* Bueno-Soria & Holzenthal, 2003

*Metrichiatriquetra* Bueno-Soria & Holzenthal, 2003

*Metrichiatruncata* Bueno-Soria & Holzenthal, 2003

*Nothotrichiamunozi* Holzenthal & Harris, 2002

*Nothotrichiatupi* Holzenthal & Harris, 2022

*Ochrotrichiaaffinis* Bueno-Soria & Holzenthal, 2004

*Ochrotrichiaalargada* Bueno-Soria & Holzenthal, 2004

*Ochrotrichiaamorfa* Bueno-Soria & Holzenthal, 2004

*Ochrotrichiaassita* Bueno-Soria & Holzenthal, 2004

*Ochrotrichiaavicula* Bueno-Soria & Holzenthal, 2008

*Ochrotrichiaavis* Bueno-Soria & Holzenthal, 1998

*Ochrotrichiabractea* Bueno-Soria & Holzenthal, 2004

*Ochrotrichiacatarina* Bueno-Soria & Holzenthal, 2004

*Ochrotrichiacitra* Bueno-Soria & Holzenthal, 2004

*Ochrotrichiacompacta* Bueno-Soria & Holzenthal, 2004

*Ochrotrichiaconformalis* Bueno-Soria & Holzenthal, 2008

*Ochrotrichiacurvata* Bueno-Soria & Holzenthal, 2004

*Ochrotrichiacuspidata* Bueno-Soria & Holzenthal, 2004

*Ochrotrichiadelgada* Bueno-Soria & Holzenthal, 2004

*Ochrotrichiadulcea* Bueno-Soria & Holzenthal, 1998

*Ochrotrichiaindefinida* Bueno-Soria & Holzenthal, 2004

*Ochrotrichiainvoluta* Bueno-Soria & Holzenthal, 2004

*Ochrotrichiaixtlahuaca* Bueno-Soria & Holzenthal, 2004

*Ochrotrichiajolandae* Bueno-Soria & Holzenthal, 2008

*Ochrotrichialeona* Bueno-Soria & Holzenthal, 2004

*Ochrotrichialongispina* Bueno-Soria & Holzenthal, 2004

*Ochrotrichiamembrana* Bueno-Soria & Holzenthal, 1998

*Ochrotrichiapatulosa* Wasmund & Holzenthal, 2007

*Ochrotrichiaquasi* Bueno-Soria & Holzenthal, 2008

*Ochrotrichiaquebrada* Bueno-Soria & Holzenthal, 1998

*Ochrotrichiaquinealensis* Bueno-Soria & Holzenthal, 1998

*Ochrotrichiaramona* Bueno-Soria & Holzenthal, 1998

*Ochrotrichiaregiomontana* Bueno-Soria & Holzenthal, 2004

*Ochrotrichiasilva* Bueno-Soria & Holzenthal, 1998

*Ochrotrichiaspina* Bueno-Soria & Holzenthal, 2004

*Ochrotrichiaspinula* Bueno-Soria & Holzenthal, 2004

*Ochrotrichiaspira* Thomson & Holzenthal, 2012

*Ochrotrichiaunicornia* Bueno-Soria & Holzenthal, 2004

*Ochrotrichiavieja* Bueno-Soria & Holzenthal, 1998

*Ochrotrichiayavesia* Bueno-Soria & Holzenthal, 2004

*Orinocotrichiacalcariga* Harris, Flint & Holzenthal, 2002

*Oxyethiraapinolada* Holzenthal & Harris, 1992

*Oxyethirabettyae* Thomson & Holzenthal, 2012

*Oxyethiracuernuda* Holzenthal & Harris, 1992

*Oxyethiraculebra* Holzenthal & Harris, 1992

*Oxyethiraespinada* Holzenthal & Harris, 1992

*Oxyethirahilosa* Holzenthal & Harris, 1992

*Oxyethiraitascae* Monson & Holzenthal, 1993

*Oxyethirakingi* Holzenthal & Kelley, 1983

*Oxyethiraquiramae* Thomson & Holzenthal, 2012

*Oxyethirarareza* Holzenthal & Harris, 1992

*Oxyethiraredunca* Thomson & Holzenthal, 2012

*Oxyethirasencilla* Holzenthal & Harris, 1992

*Oxyethirasierruca* Holzenthal & Harris, 1992

*Oxyethiratica* Holzenthal & Harris, 1992

*Rhyacopsychebenwa* Wasmund & Holzenthal, 2007

*Rhyacopsychebulbosa* Wasmund & Holzenthal, 2007

*Rhyacopsychecolei* Wasmund & Holzenthal, 2007

*Rhyacopsychecolombiana* Wasmund & Holzenthal, 2007

*Rhyacopsychecolubrinosa* Wasmund & Holzenthal, 2007

*Rhyacopsychedikrosa* Wasmund & Holzenthal, 2007

*Rhyacopsycheflinti* Wasmund & Holzenthal, 2007

*Rhyacopsychehasta* Wasmund & Holzenthal, 2007

*Rhyacopsycheintraspira* Wasmund & Holzenthal, 2007

*Rhyacopsycherhamphisa* Wasmund & Holzenthal, 2007

*Rhyacopsychetanylobosa* Wasmund & Holzenthal, 2007

*Tizatetrichiacostaricensis* Harris, Flint & Holzenthal, 2002

*Tupiniquintrichiaprocera* Thomson & Holzenthal, 2015

### Lepidostomatidae

*Lepidostomachiriquiense* Holzenthal & Strand, 1992

*Lepidostomaectopium* Holzenthal & Strand, 1992

*Lepidostomapolylepidum* Holzenthal & Strand, 1992

*Lepidostomatapanti* Holzenthal & Strand, 1992

*Lepidostomaxolotl* Holzenthal & Strand, 1992

### Leptoceridae

*Adicellauwuensis* Andersen & Holzenthal, 2002

*Amazonatolicahamadae* Holzenthal & Oliveira Pes, 2004

*Amphoropsychearagua* Holzenthal, 1985

*Amphoropsycheayura* Holzenthal, 1985

*Amphoropsychecarchi* Rázuri-Gonzales, Holzenthal & Ríos-Touma, 2017

*Amphoropsychecauca* Holzenthal, 1985

*Amphoropsychechoco* Holzenthal, 1985

*Amphoropsycheflinti* Holzenthal, 1985

*Amphoropsychematsigenka* Rázuri-Gonzales, Holzenthal & Ríos-Touma, 2017

*Amphoropsychenapo* Holzenthal, 1985

*Amphoropsychequebrada* Holzenthal, 1985

*Amphoropsychereal* Holzenthal, 2016

*Amphoropsycherefugia* Holzenthal, 1985

*Amphoropsychespinifera* Holzenthal, 1986

*Amphoropsychestellata* Holzenthal, 1985

*Amphoropsychetandayapa* Holzenthal & Rázuri-Gonzales, 2011

*Atanatolicaacuminata* Holzenthal, 1988

*Atanatolicaandina* Rázuri-Gonzales, Holzenthal & Ríos-Touma, 2018

*Atanatolicaangulata* Rázuri-Gonzales, Holzenthal & Ríos-Touma, 2018

*Atanatolicaaurea* Holzenthal, 1988

*Atanatolicacaldas* Holzenthal, 1988

*Atanatolicachoco* Holzenthal, 1988

*Atanatolicacotopaxi* Holzenthal, 1988

*Atanatolicacurvata* Rázuri-Gonzales, Holzenthal & Ríos-Touma, 2018

*Atanatolicadecouxi* Rázuri-Gonzales, Holzenthal & Ríos-Touma, 2018

*Atanatolicaflinti* Holzenthal, 2018

*Atanatolicamanabi* Holzenthal, 2018

*Atanatolicamoselyi* Denning & Holzenthal, 1988

*Atanatolicamuyupampa* Holzenthal, 2018

*Atanatolicanigra* Holzenthal, 2018

*Atanatolicanivea* Holzenthal, 2018

*Atanatolicapanamensis* Holzenthal, 2018

*Atanatolicapenai* Holzenthal, 2018

*Atanatolicazongo* Holzenthal, 2018

*Fernandoschmidiaamudita* Holzenthal & Andersen, 2007

*Fernandoschmidiaaramaniya* Holzenthal & Andersen, 2007

*Grumichellablahniki* Calor & Holzenthal, 2016

*Grumichellaboraceia* Calor & Holzenthal, 2016

*Grumichellacressae* Calor & Holzenthal, 2016

*Grumichellajureia* Calor & Holzenthal, 2016

*Grumichellaleccii* Calor & Holzenthal, 2016

*Grumichellamuelleri* Calor & Holzenthal, 2016

*Grumichellapaprockii* Calor & Holzenthal, 2016

*Grumichellaparati* Calor & Holzenthal, 2016

*Grumichellatrujilloi* Calor & Holzenthal, 2016

*Nectopsycheexophthalma* Holzenthal, 1995

*Nectopsychemonticola* Holzenthal, 1995

*Nectopsychenavasi* Holzenthal, 2000

*Nectopsycheonyx* Holzenthal, 1995

*Nectopsycheortizi* Holzenthal, 1995

*Nectopsychepadrenavasi* Holzenthal, 2000

*Nectopsychetapanti* Holzenthal, 1995

*Nectopsychetuanis* Holzenthal, 1995

*Nectopsycheutleyorum* Holzenthal, 1995

*Neoathripsodesanomalus* Holzenthal, 1989

*Neoatriplectidesfroehlichi* Holzenthal, 1997

*Notalinabrasiliana* Holzenthal, 1986

*Notalinacipo* Holzenthal, 1986

*Notalinafroehlichi* Calor & Holzenthal, 2006

*Notalinahamiltoni* Holzenthal, 1986

*Notalinamatthiasi* Holzenthal, 1986

*Notalinamorsei* Holzenthal, 1986

*Notalinananay* Holzenthal, 1986

*Notalinapaulista* Calor & Holzenthal, 2006

*Notalinaroraima* Holzenthal, 1986

*Oecetisacciptrina* Blahnik & Holzenthal, 2014

*Oecetisacuticlasper* Quinteiro & Holzenthal, 2017

*Oecetisagosta* Blahnik & Holzenthal, 2014

*Oecetisangularis* Blahnik & Holzenthal, 2014

*Oecetisapache* Blahnik & Holzenthal, 2014

*Oecetisbidigitata* Quinteiro & Holzenthal, 2017

*Oecetisblahniki* Quinteiro & Holzenthal, 2017

*Oecetiscalori* Quinteiro & Holzenthal, 2017

*Oecetiscampana* Blahnik & Holzenthal, 2014

*Oecetiscarinata* Quinteiro & Holzenthal, 2017

*Oecetiscassicoleata* Quinteiro & Holzenthal, 2017

*Oecetisconstricta* Blahnik & Holzenthal, 2014

*Oecetisflinti* Quinteiro & Holzenthal, 2017

*Oecetisgibbosa* Quinteiro & Holzenthal, 2017

*Oecetishastapulla* Quinteiro & Holzenthal, 2017

*Oecetishoughtoni* Blahnik & Holzenthal, 2014

*Oecetislicina* Quinteiro & Holzenthal, 2017

*Oecetismachaera* Quinteiro & Holzenthal, 2017

*Oecetismaritza* Blahnik & Holzenthal, 2014

*Oecetismexicana* Blahnik & Holzenthal, 2014

*Oecetispatula* Blahnik & Holzenthal, 2014

*Oecetispertica* Quinteiro & Holzenthal, 2017

*Oecetisplenuspinosa* Quinteiro & Holzenthal, 2017

*Oecetisprotrusa* Blahnik & Holzenthal, 2014

*Oecetisquasipunctata* Quinteiro & Holzenthal, 2017

*Oecetissordida* Blahnik & Holzenthal, 2014

*Oecetistumida* Blahnik & Holzenthal, 2014

*Oecetisuncata* Blahnik & Holzenthal, 2014

*Oecetisverrucula* Blahnik & Holzenthal, 2014

*Osflintiamanu* Calor & Holzenthal, 2008

*Setodesarenatus* Holzenthal, 1982

*Setodesdixiensis* Holzenthal, 1982

*Tagalopsycheapratita* Holzenthal & Andersen, 2007

*Tagalopsychejolandae* Holzenthal & Andersen, 2007

*Tagalopsychekjaerandseni* Holzenthal & Andersen, 2007

*Tagalopsycheudagama* Holzenthal & Andersen, 2007

*Triaenodesacanthus* Holzenthal & Andersen, 2004

*Triaenodesakosua* Andersen & Holzenthal, 2001

*Triaenodesakua* Andersen & Holzenthal, 2002

*Triaenodesamma* Andersen & Holzenthal, 2001

*Triaenodesbulupendek* Andersen & Holzenthal, 1999

*Triaenodeschirripo* Holzenthal & Andersen, 2004

*Triaenodesclauseni* Holzenthal & Andersen, 2004

*Triaenodescuyotenango* Holzenthal & Andersen, 2004

*Triaenodesflintorum* Holzenthal & Andersen, 2004

*Triaenodesguadaloupe* Holzenthal & Andersen, 2004

*Triaenodeshansi* Pauls, Holzenthal & Ngera, 2010

*Triaenodeshodgesi* Holzenthal & Andersen, 2004

*Triaenodeshornitos* Holzenthal & Andersen, 2004

*Triaenodeskilambe* Holzenthal & Andersen, 2004

*Triaenodeskofi* Andersen & Holzenthal, 2002

*Triaenodeskwabena* Andersen & Holzenthal, 2002

*Triaenodeskwadwo* Andersen & Holzenthal, 2001

*Triaenodeskwaku* Andersen & Holzenthal, 2002

*Triaenodeskwame* Andersen & Holzenthal, 2002

*Triaenodeskwasi* Andersen & Holzenthal, 2002

*Triaenodesmalickyi* Pauls, Holzenthal & Ngera, 2010

*Triaenodesmexicanus* Holzenthal & Andersen, 2004

*Triaenodesmoncho* Holzenthal & Andersen, 2004

*Triaenodesmorai* Holzenthal & Andersen, 2004

*Triaenodesnicaraguensis* Holzenthal & Andersen, 2004

*Triaenodesoaxacensis* Holzenthal & Andersen, 2004

*Triaenodestajo* Holzenthal & Andersen, 2004

*Triaenodestalamanca* Holzenthal & Andersen, 2004

*Triaenodestapanti* Holzenthal & Andersen, 2004

*Triaenodestico* Holzenthal & Andersen, 2004

*Triaenodestuxtlensis* Holzenthal & Andersen, 2004

*Triaenodeswoldai* Holzenthal & Andersen, 2004

*Triplectideschilensis* Holzenthal, 1988

*Triplectidesflintorum* Holzenthal, 1988

*Triplectidesmisionensis* Holzenthal, 1988

*Triplectidesneblinus* Holzenthal, 1988

*Triplectidesneotropicus* Holzenthal, 1988

*Triplectidesnevadus* Holzenthal, 1988

*Triplectidestepui* Holzenthal, 1988

*Triplectidesultimus* Holzenthal, 1988

### Philopotamidae

*Chimarraamica* Blahnik & Holzenthal, 1992

*Chimarraantheae* Blahnik, Holzenthal & Huisman, 2009

*Chimarracaduca* Blahnik, Holzenthal & Huisman, 2009

*Chimarracalori* Blahnik & Holzenthal, 2012

*Chimarracauca* Blahink & Holzenthal, 2012

*Chimarrachanchuluni* Blahnik, Holzenthal & Huisman, 2009

*Chimarracolmillo* Blahnik & Holzenthal, 1992

*Chimarracurvipenis* Blahnik & Holzenthal, 2012

*Chimarracuspidata* Blahnik, Holzenthal & Huisman, 2009

*Chimarracygnus* Blahnik, Holzenthal & Huisman, 2009

*Chimarradanumensis* Blahnik, Holzenthal & Huisman, 2009

*Chimarradejongi* Blahnik, Holzenthal & Huisman, 2009

*Chimarradenticula* Blahnik, Holzenthal & Huisman, 2009

*Chimarradesirae* Blahnik & Holzenthal, 2012

*Chimarradevogeli* Blahnik, Holzenthal & Huisman, 2009

*Chimarradrepane* Blahnik, Holzenthal & Huisman, 2009

*Chimarrafuilianae* Blahnik, Holzenthal & Huisman, 2009

*Chimarraguanacasteca* Blahnik & Holzenthal, 1992

*Chimarragyrospina* Blahnik, Holzenthal & Huisman, 2009

*Chimarrainchoata* Blahnik & Holzenthal, 2012

*Chimarrajannekae* Blahnik, Holzenthal & Huisman, 2009

*Chimarrajanzeni* Blahnik & Holzenthal, 1992

*Chimarrajemima* Blahnik & Holzenthal, 1992

*Chimarrakarlijnae* Blahnik, Holzenthal & Huisman, 2009

*Chimarrakinabaluensis* Blahnik, Holzenthal & Huisman, 2009

*Chimarralambi* Blahnik, Holzenthal & Huisman, 2009

*Chimarralata* Blahnik & Holzenthal, 1992

*Chimarralatiforceps* Blahnik & Holzenthal, 2012

*Chimarraliwaguensis* Blahnik, Holzenthal & Huisman, 2009

*Chimarralongiterga* Blahnik & Holzenthal, 1992

*Chimarramunozi* Blahnik & Holzenthal, 1992

*Chimarranicehuh* Blahnik & Holzenthal, 2012

*Chimarranoloyan* Blahnik, Holzenthal & Huisman, 2009

*Chimarranoohi* Blahnik, Holzenthal & Huisman, 2009

*Chimarraonchyrhina* Blahnik & Holzenthal, 2012

*Chimarraparaortiziana* Blahnik & Holzenthal, 1992

*Chimarrapeineta* Blahnik & Holzenthal, 1992

*Chimarraphillipsae* Blahnik, Holzenthal & Huisman, 2009

*Chimarraphysanoton* Blahnik, Holzenthal & Huisman, 2009

*Chimarrapollex* Blahnik & Holzenthal, 1992

*Chimarrapreapicalis* Blahnik, Holzenthal & Huisman, 2009

*Chimarrascolops* Blahnik, Holzenthal & Huisman, 2009

*Chimarrasilausilau* Blahnik, Holzenthal & Huisman, 2009

*Chimarrasinitorum* Blahnik, Holzenthal & Huisman, 2009

*Chimarrasolisi* Blahnik & Holzenthal, 1992

*Chimarrasoroa* Blahnik & Holzenthal, 2012

*Chimarrastenodactylus* Blahnik, Holzenthal & Huisman, 2009

*Chimarrasunima* Blahnik & Holzenthal, 2012

*Chimarravantoli* Blahnik, Holzenthal & Huisman, 2009

*Chimarravanwelzeni* Blahnik, Holzenthal & Huisman, 2009

*Chimarraventritropis* Blahnik, Holzenthal & Huisman, 2009

*Chimarravirgencita* Blahnik & Holzenthal, 1992

*Chimarraxiphosella* Blahnik, Holzenthal & Huisman, 2009

*Chimarrayanura* Blahnik & Holzenthal, 1992

*Chimarrhodellachoco* Holzenthal, Blahnik & Ríos-Touma, 2018

*Chimarrhodellacostaricensis* Blahnik & Holzenthal, 1992

*Chimarrhodellaflinti* Blahnik & Holzenthal, 1992

*Chimarrhodellapilcopata* Blahnik & Holzenthal, 1992

*Chimarrhodellatapanti* Blahnik & Holzenthal, 1992

*Chimarrhodellatobagoensis* Blahnik & Holzenthal, 1992

*Hydrobiosellaandina* Holzenthal, Blahnik & Ríos-Touma, 2018

*Wormaldiaandrea* Munoz-Quesada & Holzenthal, 2015

*Wormaldiaanhelitus* Munoz-Quesada & Holzenthal, 2015

*Wormaldiaaraujoi* Munoz-Quesada & Holzenthal, 2015

*Wormaldiaaymara* Munoz-Quesada & Holzenthal, 2015

*Wormaldiabarbai* Munoz-Quesada & Holzenthal, 2015

*Wormaldiabirneyi* Munoz-Quesada & Holzenthal, 2008

*Wormaldiabolivari* Munoz-Quesada & Holzenthal, 2015

*Wormaldiaboteroi* Munoz-Quesada & Holzenthal, 2015

*Wormaldiabuenorum* Munoz-Quesada & Holzenthal, 2015

*Wormaldiacalderonae* Munoz-Quesada & Holzenthal, 2015

*Wormaldiachrismark* Munoz-Quesada & Holzenthal, 2015

*Wormaldiaclauseni* Munoz-Quesada & Holzenthal, 2008

*Wormaldiacontrerasi* Munoz-Quesada & Holzenthal, 2015

*Wormaldiacornuta* Bueno-Soria & Holzenthal, 1986

*Wormaldiadachiardiorum* Munoz-Quesada & Holzenthal, 2015

*Wormaldiaeberhardi* Munoz-Quesada & Holzenthal, 2015

*Wormaldiaflinti* Munoz-Quesada & Holzenthal, 2015

*Wormaldiafrancovilla* Munoz-Quesada & Holzenthal, 2015

*Wormaldiafredycarol* Munoz-Quesada & Holzenthal, 2015

*Wormaldiagallardoi* Munoz-Quesada & Holzenthal, 2015

*Wormaldiagonzalezae* Munoz-Quesada & Holzenthal, 2015

*Wormaldiahedamafera* Munoz-Quesada & Holzenthal, 2015

*Wormaldiaimberti* Munoz-Quesada & Holzenthal, 2015

*Wormaldiaimbrialis* Holzenthal, Blahnik & Ríos-Touma, 2018

*Wormaldiainca* Munoz-Quesada & Holzenthal, 2015

*Wormaldiaisela* Munoz-Quesada & Holzenthal, 2015

*Wormaldiajuarox* Munoz-Quesada & Holzenthal, 2015

*Wormaldialauglo* Munoz-Quesada & Holzenthal, 2015

*Wormaldialuma* Bueno-Soria & Holzenthal, 1986

*Wormaldiamachadorum* Munoz-Quesada & Holzenthal, 2015

*Wormaldiamaesi* Munoz-Quesada & Holzenthal, 2015

*Wormaldiamenchuae* Munoz-Quesada & Holzenthal, 2015

*Wormaldiamonsonorum* Munoz-Quesada & Holzenthal, 2015

*Wormaldianavarroae* Munoz-Quesada & Holzenthal, 2015

*Wormaldiapaprockevi* Munoz-Quesada & Holzenthal, 2015

*Wormaldiasaboriorum* Munoz-Quesada & Holzenthal, 2015

*Wormaldiatarasca* Bueno-Soria & Holzenthal, 1986

*Wormaldiatocajoma* Munoz-Quesada & Holzenthal, 2015

*Wormaldiatrondi* Munoz-Quesada & Holzenthal, 2015

*Wormaldiatupacamara* Munoz-Quesada & Holzenthal, 2015

*Wormaldiazunigae* Munoz-Quesada & Holzenthal, 2015

*Wormaldiazunigarceorum* Munoz-Quesada & Holzenthal, 2015

### Polycentropodidae

*Cernotinaantonina* Holzenthal & de Almeida, 2003

*Cernotinalazzarii* Holzenthal & de Almeida, 2003

*Cernotinatiputini* Camargos, Ríos-Touma & Holzenthal, 2017

*Cernotinawaorani* Camargos, Ríos-Touma & Holzenthal, 2017

*Polycentropusacinaciformis* Hamilton & Holzenthal, 2011

*Polycentropusamphirhamphus* Hamilton & Holzenthal, 2011

*Polycentropusancistrus* Hamilton & Holzenthal, 2011

*Polycentropusboraceia* Hamilton & Holzenthal, 2011

*Polycentropuscaaete* Hamilton & Holzenthal, 2011

*Polycentropuscachoeira* Hamilton & Holzenthal, 2011

*Polycentropuscarioca* Hamilton & Holzenthal, 2011

*Polycentropuscarolae* Hamilton & Holzenthal, 2011

*Polycentropuscheliceratus* Hamilton & Holzenthal, 2011

*Polycentropuscipoensis* Hamilton & Holzenthal, 2011

*Polycentropuscressae* Hamilton & Holzenthal, 2005

*Polycentropusfasthi* Holzenthal & Hamilton, 1988

*Polycentropusfluminensis* Hamilton & Holzenthal, 2011

*Polycentropusfortispinus* Holzenthal & Hamilton, 1988

*Polycentropusfroehlichi* Hamilton & Holzenthal, 2011

*Polycentropusgalharada* Hamilton & Holzenthal, 2011

*Polycentropusgraciosa* Hamilton & Holzenthal, 2011

*Polycentropusinusitatus* Hamilton & Holzenthal, 2011

*Polycentropusitatiaia* Hamilton & Holzenthal, 2011

*Polycentropusminero* Hamilton & Holzenthal, 2011

*Polycentropusneblinensis* Hamilton & Holzenthal, 2005

*Polycentropusnebulosus* Holzenthal & Hamilton, 1988

*Polycentropuspaprockii* Hamilton & Holzenthal, 2011

*Polycentropusquadricuspidis* Hamilton & Holzenthal, 2005

*Polycentropusrosalysae* Hamilton & Holzenthal, 2011

*Polycentropussantateresae* Hamilton & Holzenthal, 2011

*Polycentropussilex* Hamilton & Holzenthal, 2005

*Polycentropussoniae* Hamilton & Holzenthal, 2011

*Polycentropusthaxtoni* Hamilton & Holzenthal, 1986

*Polycentropustripui* Hamilton & Holzenthal, 2011

*Polycentropusurubici* Holzenthal & De Almeida, 2003

*Polycentropusverruculus* Hamilton & Holzenthal, 2011

*Polycentropusvirginiae* Hamilton & Holzenthal, 2011

*Polycentropusvolcanus* Holzenthal & Hamilton, 1988

*Polycentropuszurqui* Holzenthal & Hamilton, 1988

*Polyplectropusadamsae* Chamorro & Holzenthal, 2010

*Polyplectropusalatespinus* Chamorro & Holzenthal, 2010

*Polyplectropusamazonicus* Chamorro & Holzenthal, 2010

*Polyplectropusandinensis* Chamorro & Holzenthal, 2010

*Polyplectropusbeccus* Hamilton & Holzenthal, 2005

*Polyplectropusblahniki* Chamorro & Holzenthal, 2010

*Polyplectropusbolivianus* Chamorro & Holzenthal, 2010

*Polyplectropusbrasilensis* Chamorro & Holzenthal, 2010

*Polyplectropusbrborichorum* Chamorro & Holzenthal, 2010

*Polyplectropusclauseni* Chamorro-Lacayo & Holzenthal, 2004

*Polyplectropuscolombianus* Chamorro & Holzenthal, 2010

*Polyplectropuscorniculatus* Chamorro & Holzenthal, 2010

*Polyplectropuscressae* Chamorro & Holzenthal, 2010

*Polyplectropuscuzcoensis* Chamorro & Holzenthal, 2010

*Polyplectropusecuadoriensis* Chamorro & Holzenthal, 2010

*Polyplectropusexilis* Chamorro-Lacayo & Holzenthal, 2004

*Polyplectropusflintorum* Chamorro & Holzenthal, 2010

*Polyplectropusgaesum* Chamorro & Holzenthal, 2010

*Polyplectropusguyanae* Chamorro & Holzenthal, 2010

*Polyplectropushollyae* Chamorro & Holzenthal, 2010

*Polyplectropushymenochilus* Chamorro-Lacayo & Holzenthal, 2004

*Polyplectropushystricosus* Chamorro & Holzenthal, 2010

*Polyplectropusinsularis* Chamorro & Holzenthal, 2010

*Polyplectropusjuliae* Chamorro & Holzenthal, 2010

*Polyplectropuskanukarum* Chamorro & Holzenthal, 2010

*Polyplectropuskylistos* Chamorro-Lacayo & Holzenthal, 2004

*Polyplectropusmaculatus* Chamorro & Holzenthal, 2010

*Polyplectropusmanuensis* Chamorro & Holzenthal, 2010

*Polyplectropusmatatlanticus* Chamorro & Holzenthal, 2010

*Polyplectropusminensium* Chamorro & Holzenthal, 2010

*Polyplectropusnovafriburgensis* Chamorro & Holzenthal, 2010

*Polyplectropusparadelphae* Chamorro-Lacayo & Holzenthal, 2004

*Polyplectropusperpendicularis* Chamorro-Lacayo & Holzenthal, 2004

*Polyplectropusperuvianus* Chamorro & Holzenthal, 2010

*Polyplectropuspetrae* Chamorro & Holzenthal, 2010

*Polyplectropuspratherae* Chamorro & Holzenthal, 2010

*Polyplectropusprofaupar* Holzenthal & De Almeida, 2003

*Polyplectropuspuyoensis* Chamorro & Holzenthal, 2010

*Polyplectropusrobertsonae* Chamorro & Holzenthal, 2010

*Polyplectropusrodmani* Chamorro & Holzenthal, 2010

*Polyplectropusrondoniensis* Chamorro & Holzenthal, 2010

*Polyplectropustragularius* Chamorro & Holzenthal, 2010

*Polyplectropustripunctatum* Chamorro & Holzenthal, 2010

*Polyplectropusvenezolanus* Chamorro & Holzenthal, 2010

*Polyplectropuswoldai* Chamorro & Holzenthal, 2010

*Polyplectropusyolandae* Chamorro-Lacayo & Holzenthal, 2004

*Polyplectropuszamoranoensis* Chamorro & Holzenthal, 2010

*Polyplectropuszuliae* Chamorro & Holzenthal, 2010

### Sericostomatidae

*Notidobiellaamazoniana* Holzenthal & Blahnik, 2011

*Notidobiellabrasiliana* Holzenthal & Blahnik, 2011

*Notidobiellaecuadorensis* Holzenthal & Blahnik, 2011

### Xiphocentronidae

*Machairocentronchorotegae* Vilarino & Holzenthal, 2020

*Machairocentroneugeniarguedasae* Vilarino & Holzenthal, 2020

*Machairocentronkalinae* Vilarino & Holzenthal, 2020

*Xiphocentronmoncho* Munoz-Quesada & Holzenthal, 1997

### Species named after R.W. Holzenthal

*Alisotrichiaholzenthali* Santos, 2011

*Alterosaholzenthali* Blahnik, 2005

*Anchitrichiaholzenthali* Oláh & Flint, 2012

*Chimarraholzenthali* Lago & Harris, 1987

*Corydalusralphi* Martins, Azevêdo, Hamada & Contreras, 2022

*Helicopsycheholzenthali* Johanson, 2003

*Helicopsycheralphi* Cavalcante-Silva, Pereira & Calor, 2022

*Hydroptilaholzenthali* Sykora & Harris, 1994

*Kisauraholzenthali* Phander & Saini, 2014

*Leucotrichiaholzenthali* Thomson, Armitage & Harris, 2022

*Mariliaholzenthali* Bueno-Soria & Rojas-Ascencio, 2004

*Metalypeholzenthali* (Schmid, 1997)

*Neoathripsodesholzenthali* Dias, Quinteiro & Calor, 2015

Notalina (Neonotalina) ralphi Silva Pereira, Oliveira, Robson Desidério, Calor & Hamada, 2022

*Phylloicusholzenthali* Prather, 2003

*Polycentropusholzenthali* Bueno-Soria & Hamilton, 1986

*Silvataresholzenthali* Rázuri-Gonzales, Ngera & Pauls, 2022

*Smicrideaholzenthali* Flint & Denning, 1989

*Smicridearalphi* Almeida & Flint, 2002
